# Protein-Nanoparticle Interactions Govern the Interfacial Behavior of Polymeric Nanogels: Study of Protein Corona Formation at the Air/Water Interface

**DOI:** 10.3390/ijms24032810

**Published:** 2023-02-01

**Authors:** Federico Traldi, Pengfei Liu, Inês Albino, Lino Ferreira, Ali Zarbakhsh, Marina Resmini

**Affiliations:** 1 Department of Chemistry, SPCS, Queen Mary University of London, London E1 4NS, UK; 2 CNC-Center for Neuroscience and Cell Biology, University of Coimbra, UC, Biotech Parque Tecnológico de Cantanhede, 3060-197 Coimbra, Portugal; 3 Faculty of Medicine, University of Coimbra, 3060-197 Coimbra, Portugal

**Keywords:** surface tensiometry, protein corona, nanogels, interfacial behavior, neutron reflectivity

## Abstract

Biomedical applications of nanoparticles require a fundamental understanding of their interactions and behavior with biological interfaces. Protein corona formation can alter the morphology and properties of nanomaterials, and knowledge of the interfacial behavior of the complexes, using in situ analytical techniques, will impact the development of nanocarriers to maximize uptake and permeability at cellular interfaces. In this study we evaluate the interactions of acrylamide-based nanogels, with neutral, positive, and negative charges, with serum-abundant proteins albumin, fibrinogen, and immunoglobulin G. The formation of a protein corona complex between positively charged nanoparticles and albumin is characterized by dynamic light scattering, circular dichroism, and surface tensiometry; we use neutron reflectometry to resolve the complex structure at the air/water interface and demonstrate the effect of increased protein concentration on the interface. Surface tensiometry data suggest that the structure of the proteins can impact the interfacial properties of the complex formed. These results contribute to the understanding of the factors that influence the bio-nano interface, which will help to design nanomaterials with improved properties for applications in drug delivery.

## 1. Introduction

Progress in nanotechnology has led to the development of nanoparticles (NPs) with a wide range of applications in sensing [1], catalysis [2], and nanomedicine [3]. The use of nanomaterials as drug delivery systems, in particular, offers potential advantages such as improved stability and solubility of drugs [4], promoted drug transport across membranes [5], and site-specific targeting of therapeutics [6]. However, the application of NPs in clinical settings remains challenging, due to the potential issues with their long-term toxicity [7], and interactions with biomolecules such as proteins [8,9], lipids [10,11], sugars [12], and oligonucleotides [13]. In particular, protein corona formation has been shown to alter the morphology and structure of NPs, impacting on bioavailability [14,15], toxicity [16,17], and targeting [18,19,20,21,22], all factors that can affect their applications. However, recent data have highlighted how protein corona formation can also be tailored to become an advantage. Kim et al. modified the chemistry of DNA nanostructures to form an apolipoprotein-enriched protein corona, able to target liver tissue for the treatment of fibrosis [23]. Mosquera et al. used an external stimulus to control protein corona formation on gold NPs, to influence their cellular internalization in vitro [14]. Moreover, the study of nanoparticle-protein interactions has led to interesting applications in the development of sensors [24]. Thus, understanding how the chemistry and morphology of nanomaterials can be used to control the formation of protein corona is essential for developing successful applications. 

Studies of protein corona formation have traditionally involved the isolation of NPs-protein complexes prior to analysis [25,26], resulting in only the strongest interactions being evaluated, using a broad range of analytical techniques [27]. This is often referred to as the hard corona. Although the data acquired by this approach is very useful, especially knowing the composition of the proteins involved in the complex, it has been demonstrated that the necessary isolation step often results in the alteration of the structure and composition of the complex [28,29]. There is considerable interest now in studying the phenomena of protein corona formation in greater depth, focusing in particular on the weaker interactions occurring in the solution between the NPs and proteins. When evaluating the isolated complex, very limited data can be obtained on the weaker NPs-protein associations occurring in the solution; recently these interactions have been shown to play an important role in influencing the morphology and structure of the outer layer of the complexes [30,31,32], hence impacting the outcome of any applications, especially in diagnostics and in vivo. The characterization of these phenomena is now a priority; to understand the behavior of NPs, and the use of different analytical approaches is important. 

Recently, the focus has been on techniques that allow the evaluation of the complex formation in the solution (‘in situ’ techniques), such as dynamic light scattering (DLS) [33], isothermal titration calorimetry (ITC) [34], fluorescence correlation spectroscopy (FCS) [35], fluorescence quenching [36], nuclear magnetic resonance (NMR) [37], electron microscopy techniques [38,39], super resolution microscopy [40,41], small-angle neutron scattering [42], and dark-field microscopy [43,44]. Some interesting results have been reported, although the complexity of the mixtures still limits the information available with a single method. Recently, the use of drop analysis to measure changes in the interfacial behavior of silica NPs with bovine serum albumin (BSA) was reported [45], although it provides lower sensitivity.

There is a growing interest in broadening the range of techniques used for the characterization of protein corona, in particular its impact on the interfacial behavior of NPs. This is important, in view of all the applications requiring the interactions of NPs with biological interfaces, such as membranes and cell surfaces [46]. Surface force tensiometry is an analytical technique that allows the study of interfacial properties of colloidal solutions; we have used it extensively to characterize the behavior of N-isopropylacrylamide (NIPAM)-based nanogels. 

Polymeric organic nanogels are widely studied as drug delivery platforms thanks to their colloidal stability, biocompatibility, efficient drug uploading and potential for stimuli responsive behaviour [47,48,49]. Our group has studied the structure and conformation of cross-linked NIPAM nanogels with a size of around 10 nm, and their potential applications in sensing [50,51], and drug delivery [52,53]. We reported our findings on the role that rigidity plays in influencing their assembly at the air/water interface [54], and how interactions with lipid mono- and multi-bilayers can impact their interfacial behavior [55,56]. Investigations of protein corona have primarily focused so far on large organic NPs, such as polystyrene [57], liposomes [58], and PLGA particles [59], or smaller inorganic ones, such as gold NPs [36,60]; however, as the physico-chemical properties of materials change with size, there is a clear need to characterize the protein corona of small polymeric NPs such as nanogels, to support their biomedical applications, especially as drug delivery systems.

In this paper, we present our study on the characterization of the in situ complex formation between acrylamide-based nanogels, neutral and charged, and proteins, including BSA, fibrinogen (Fib), and immunoglobulin G (IgG). These proteins are found in a relatively high concentration in plasma and represent major constituents of the protein corona of liposomes [61], polystyrene NPs [57,62], silica NPs [63], and metal-based NPs [64,65]. We initially evaluated the complex formation between BSA and the nanogels using DLS, circular dichroism (CD), and surface tensiometry. The structure of the complex at the air/water interface, mimicking the bio-nano interface, was resolved by neutron reflectivity (NR) studies. Surface tensiometry was finally used to evaluate the interfacial behavior of nanogels with Fib and IgG, to further expand on the range of proteins and their influence on protein corona formation.

## 2. Results and Discussion

### 2.1. Synthesis and Characterization of Nanogels

**Scheme 1:**
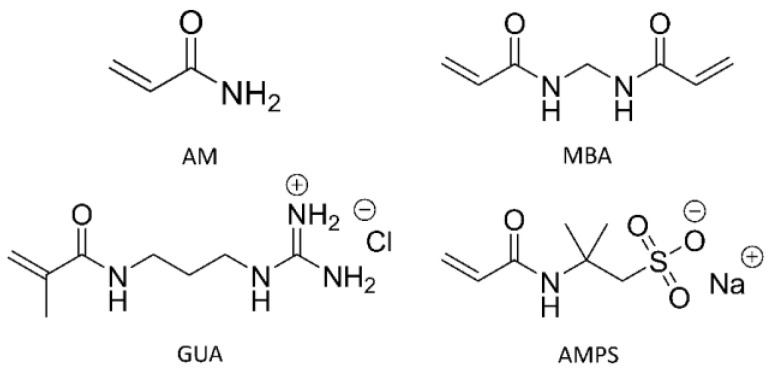
Chemical structure of monomers and crosslinker used in the synthesis of nanogels.

Three acrylamide-based nanogels were synthesized using HDRP, a method which allows the control of nanogels’ size and polydispersity without the need for surfactants [66]. Acrylamide (AM) and *N*,*N*′-methylenbisacrylamide (MBA) were chosen as a backbone monomer and a crosslinker, respectively (Scheme 1). AM was chosen because of its higher hydrophilicity, with the expectation of yielding non-active nanogels. All nanogels were synthesized with crosslinker (MBA) content fixed at 20 mol%, which is the percentage of moles of crosslinker based on the total moles of monomers in the formulation, as it was previously shown to lead to a matrix with good drug loading properties, suitable for drug delivery applications [52,54]. Charged nanogels were obtained by adding 20 mol % of GUA or AMPS to the formulation, to ensure a strong surface charge was available on the NP. 

The nanogels were obtained with high monomer conversion (>80% by ^1^H NMR) and chemical yields (>70%) as shown in Table 1, which ensures a good consistency between formulation and chemical structure of the matrix. Lower chemical yields compared to monomer conversions are often the result of the loss of low molecular weight (<3.5 kDa) polymer chains, during the purification step via dialysis. The polymerization steps are optimized to ensure high yields, which therefore minimizes the presence of short oligomers, which are not characterised as these particles are not of interest for application purposes. All three nanogels were obtained with a particle size between 5 and 10 nm, determined by DLS number distribution (Figure S1), while z-potential analysis confirmed the positive (+9.1 mV) and negative (−14.5 mV) surface charge for NG_pos_ and NG_neg_, respectively. Nanogels with a similar diameter have the capacity to minimize the impact of size on protein corona formation, making these nanogels suitable for studying the effect of surface charge and chemistry on the formation of protein corona [16,36,67]. Surface tension measurements confirmed that the nanogels presented no significant surface activity within 24 h (Figure S2), making these materials good candidates to study the use of surface tensiometry to measure their protein corona formation. 

### 2.2. Interaction of Nanogels with BSA by Dynamic Light Scattering and Surface Tensiometry

**Figure 1:**
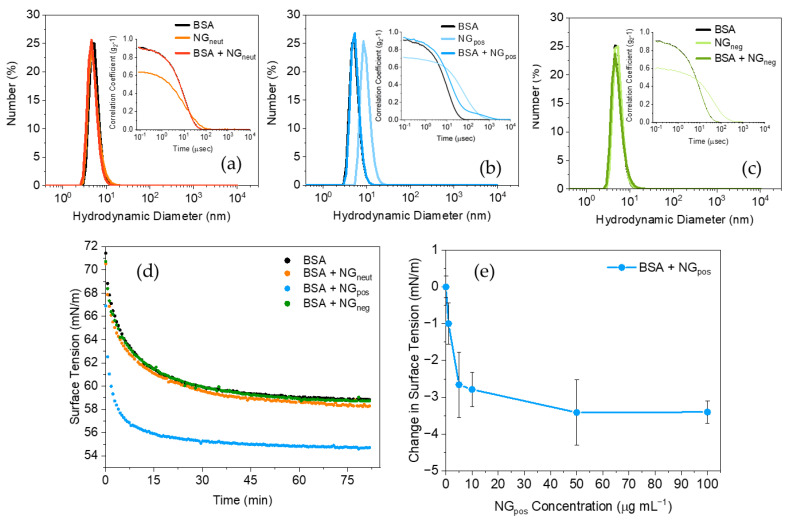
Interaction study of (**a**) neutral (NG_neut_), (**b**) positively (NG_pos_), and (**c**) negatively (NG_neg_) charged nanogels with 35 mg mL ^−1^ BSA (black) by DLS (number distribution). (**d**) Dynamic surface tensiometry of NG_neut_ (orange), NG_pos_ (blue), and NG_neg_ (green) in presence of 100 μg mL^−1^ BSA compared to the pure protein (black). (**e**) Equilibrium surface tension values for BSA (100 μg mL^−1^) with increasing concentration of NG_pos_ (0–100 μg mL^−1^). All measurements were conducted in PBS (10 mM, pH = 7.4) at 25 °C.

As albumin is the most representative protein in the serum, BSA was initially selected as a model protein to investigate its interaction with nanogels, initially using DLS. The protein was used at a concentration of 35 mg mL^−1^ to represent in vivo conditions [68]. As seen from Figure 1a–c (black line), BSA showed a Dh by number distribution of 8 nm in PBS (10 mM, pH 7.4) as found in the literature [69]. When 100 μg mL^−1^ of either NG_neut_, NG_pos_, or NG_neg_ were added to the BSA solution, no changes in the sample size distribution could be observed, thus showing no evidence of significant interaction. Interestingly, data by intensity distribution appear to suggest some changes in the size distribution of NG_pos_ (Figure S3a–c) upon addition of BSA, although interpretation of these data must be undertaken carefully due to the intensity of the light scattered from particles depending on the 6th power of their radius, thus leading to potential misrepresentation of real relative abundances of individual populations in the sample. Indeed, the peak with size around 400 nm observed in the results by intensity is likely to represent a negligible portion of the sample and is not indicative of substantial aggregation (Figure S3b). Moreover, the high concentration of BSA used in this analysis may impede the detection of the NG-BSA complex. Analysis of the protein corona on the same nanogels obtained using 0.1 mg mL^−1^ of BSA showed a similar result, with no evidence of interactions observed in the results by number (Figure S4a–c), while results by intensity indicated interactions between the protein and NG_pos_ (Figure S4d–f). Interestingly, the size of the populations identified as NG_pos_-BSA complexes increased from 21 nm to 50 nm when the concentration of BSA was increased from 0.1 to 35 mg mL^−1^, further suggesting some interaction between this nanogel and the protein. 

These data suggest that electrostatic interactions may be the main driving force for the formation of NG_pos_-BSA complexes, consistent with literature data [70,71], while other interactions such as hydrophobic are negligible in this case, as previously reported [67]. 

DLS offers a valuable tool to investigate the in situ formation of protein corona, however its low resolution limits the study of subtle interactions between nanogels and proteins of similar size [72], therefore limiting the type of conclusions that can be obtained. Moreover, although this technique provides valuable information on phenomena occurring in the bulk, it cannot evaluate the effects at the interfaces. Surface force tensiometry was therefore used to obtain additional data; this technique is only sensitive to the presence of material at the air/water interface. In this work, the use of acrylamide-based nanogels, which are not surface active, provided a simplified system where the interfacial behavior of the protein in the presence of the polymeric NPs could be evaluated.

The surface tension profile of BSA (at 100 μg mL^−1^) was measured in PBS (Figure 1d, black dots). A lower concentration of BSA was employed to optimize measurements for surface tensiometry. The protein diffused at the interface led to a decrease in surface tension to 58.6 ± 0.3 mN m^−1^, consistent with previously reported values [73,74]. The addition of NG_pos_ (100 μg mL^−1^) to the BSA solution resulted in a further drop in surface tension, to 55.0 ± 0.3 mN m^−1^ (Figure 1d) as it reached equilibrium. As NG_pos_ is not surface active, this result suggests that the NPs formed a complex with BSA in the bulk solution, followed by its adsorption at the air/water interface, significantly reducing the interfacial tension.

The addition of NG_neut_ or NG_neg_ to the BSA solution did not result in any significant changes to the surface tension of the protein, suggesting that no significant interaction was occurring, consistent with the DLS data. Complex formation due to hydrogen-bond interactions, previously reported for neutral acrylamide-based nanogels with proteins [75], was not observed; this was possibly due to the very small diameter of the NPs used, impeding the adsorption of albumin [36,67,76,77]. 

To further investigate the interfacial properties of the NG_pos_-BSA complex, the surface tension as a function of NG_pos_ concentration (0–100 μg mL^−1^) was evaluated (Figure 1e, see Figure S5a for dynamic surface tension plots). The data show a decrease in surface tension when the nanogel concentration was increased, suggesting higher concentrations of complex at the interface. With the nanogel’s concentrations higher than 50 μg mL^−1^ a plateau was reached, suggesting saturation of the surface by the NG-protein complex. The lowering of the surface tension value is evidence that the complex displays a higher affinity for the air phase, which suggests that it is more hydrophobic compared to the free BSA. Protein denaturation and associated exposure of hydrophobic amino acid residues was evaluated as a possible explanation. CD experiments (Figure S6a) revealed no significant loss of the secondary structure of BSA following the addition of NG_pos_ and complex formation. It was then concluded that the complexation might have resulted in increased hydrophobicity by a mechanism of charge screening [78], which lowered the solubility of the complex in the aqueous solution and increased its affinity for the air phase.

Overall, the data provide evidence that protein coronas not only impact the physico-chemical properties of nanogels in the bulk, but also their interfacial properties. With a view to characterizing the interfacial behaviour of the nanogels complexed with proteins, we carried out experiments using NR.

### 2.3. Protein Corona Studies by Neutron Reflectometry

The interfacial structure of BSA (4 mg mL^−1^), NG_pos_ (0.1 mg mL^−1^) and their mixture at the air/water interface was studied using NR. The corresponding normalized NR profiles in subphases of contrast-matched to air (CMAir), contrast-matched to BSA (CMBSA), and D_2_O are shown in Figure S7. All three contrasts were fitted simultaneously to a single model (solid lines, Figure S7a–c). The fitted parameters and the scattering length density (SLD) for the nanogel are presented in Table S1 and Figure S7d–f), respectively. The fitted structure of BSA consisted of a thinner upper layer of 10.3 ± 0.4 Å in thickness with ~15% of water, whilst the fully immersed lower layer was much thicker (47.0 ± 1.2 Å) but far less dense (containing ~85% of water). Given the cylindrical shape of BSA (40 × 40 × 140 Å) [79], data on the thickness of the top layer suggests significant structural deformation and flattening of the protein at the interface, which is indicative of the flexibility of the protein. As the top layer becomes close-packed and dense, a secondary layer adsorbed underneath is formed, with BSA molecules adopting a sideways-on orientation (i.e., BSA molecules adsorb with their long axes parallel to the surface). These observations are well in agreement with previous NR studies of BSA solutions at the air/water interface [80,81]. 

The three contrasts for NG_pos_ could be fitted consistently to a solvated model, where a fully immersed layer of nanogels, not in contact with the air phase, extends towards the bulk of the aqueous phase (43.0 ± 1% and 89.7 ± 0.6% solvation for the upper and lower layers, respectively, as shown in Table S1). This result supports the data obtained from surface tensiometry (Figure S2), which indicated no surface activity for NG_pos_. This is because surface tensiometry is only sensitive to the outmost surface layer [82], whereas neutrons can penetrate deeper below the interface, allowing to detect material adsorbed below the surface. This particular behaviour of NG_pos_ at the interface can be rationalized by the fact that chloride ions in PBS (0.14 M) accumulate at the air/water interface, as shown by both computational and experimental studies [83,84,85,86]. Water is a highly dielectric medium compared to air; when charged nanogels approach the interface they experience repulsive image-charge forces, thus preventing them from adsorbing at the interface [87]. However, the presence of ions from PBS causes the formation of an electric double-layer at the interface, reducing this repulsion. This leads the pendent chains of the positively nanogels to be pulled closer to the interface by the counterions, as previously described for charged particles [88], resulting in a measurable NR signal without significant decrease in surface tension of the solution. This is supported by the fact that nearly no surface-bound NG_neut_ were detected by NR (Figure S8), confirming the key role of the dielectric property of water and the electrostatic interactions between negatively charged ions, located at the interface, and positively charged polymer chains, in driving NG_pos_ to the near-surface zone. 

As can be seen from Figure S7, NR profiles of BSA and BSA-NG_pos_ complex were different from those of both pure components. Kiessig fringes (dips in the Q-range 0.05–0.10 Å^−1^) were observed for BSA-NG in CMAir contrast, indicating a well-packed structure at the interface. On the contrary, NR profiles of BSA-NG_neut_ in both D_2_O and CMAir contrasts overlapped with those of pure BSA solutions (Figure S8), demonstrating no discernible interactions. This result suggests that the electrostatic force plays a primary role in the protein-nanogel complexation. The NR data of the mixtures were fitted simultaneously to a single model for all three contrasts. The total thickness of adsorbed materials was ~180 Å (Table S1), nearly 3-fold higher than pure BSA and more than 4 times thicker than NG_pos_, providing direct evidence of the adsorption of the complex at the interface. 

Fitted scattering length density (SLD) profiles were then used to calculate the volume fractions (VF) of BSA, NG_pos_, and water as shown in Table 2. Note that the sum of VF values in the first and second layers do not exactly add up to 100%. This could be because the SLD values of BSA in all three contrasts are based on complete exchange of labile hydrogens with deuterium (D). However, this might not be always the case, since the H/D exchange rate of protons located in hydrophobic regions of the protein may be slower and thus incomplete. Secondly, the upper layer, which is in contact with the air phase, may also contain air (in addition to NGs, BSA and water) due to the formation of capillary waves at the interface. However, the overall volume fraction provides a reasonable picture of the structural conformations at the interface. Data show that the upper layer of the BSA-NG_pos_ complex at the interface was rich in BSA (~40% *v*/*v*), with only ~4% *v*/*v* NG_pos_. In contrast, the middle layer was richer in nanogels, with ~2% *v*/*v* BSA, ~15% *v*/*v* NG_pos_ and ~80% *v*/*v* water. The lower layer was mainly polymeric nanogels chains with 94% solvation. The adsorbed amount of the BSA-NG_pos_ complex was calculated to be 5.01 ± 0.57 mg m^−2^, about 1.5 times greater than the simple addition of adsorbed BSA (2.20 ± 0.07 mg m^−2^) and NG_pos_ (1.23 ± 0.04 mg m^−2^) on their own, further evidencing the presence of NG_pos_-BSA complexes at the interface. Importantly, data shows that the NG_pos_-BSA complex is oriented in such a way that BSA is exposed towards the hydrophobic air phase. This may have important implications for the interaction of nanogels with biological interfaces such as cellular membranes, which might affect the behaviour of nanogels in biological settings.

**Figure 2:**
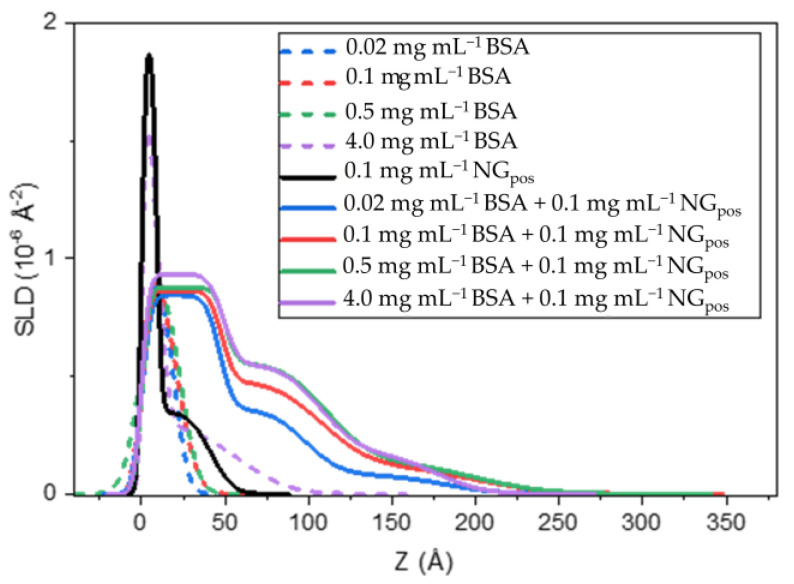
SLD profiles of BSA (dashed lines), NG_pos_ (black), and BSA-NG_pos_ mixtures (solid lines) at the air/water interface in CMAir contrast. BSA was used in concentrations between 0.02–4 mg mL^−1^, while NG_pos_ was kept at 0.1 mg mL^−1^. The NG_pos_ is made of 60 molar% d3AM—20 molar% MBA—20 molar% GUA.

We have also studied the interaction of NG_pos_ with BSA at much lower protein concentrations, including 0.1 mg mL^−1^ to match conditions employed in the analysis with surface tensiometry. The corresponding normalized NR profiles in subphase of CMAir are shown in Figure S9. The fitted parameters and the SLD for the nanogel are shown in Table S2 and Figure 2, respectively. Similar to what was reported above, the interfacial structure of the complex can be divided into three regions: a densely packed top layer, a highly solvated middle layer followed by polymeric chains diffusing to the bulk phase. The SLD profiles of the BSA solution alone do not change significantly when increasing the concentration from 0.02 to 0.5 mg ml^−1^, indicating a stable layered structure at the interface. However, in the case of BSA-NG_pos_ mixtures, the SLD value of the middle layer towards the aqueous phase increased gradually as more BSA was added to the solution. These data indicate that increasing the concentration of BSA led to more complex to form in the bulk, which resulted in more material being adsorbed at the interface on the side facing the aqueous subphase.

### 2.4. Interaction of Positive Nanogels with Fibrinogen (Fib) or Immunoglobulin G (IgG) by Dynamic Light Scattering and Surface Tensiometry

Overall, data obtained by NR confirmed the formation of NG_pos_-BSA complexes via electrostatic interactions and provided evidence of the reliability of the data obtained by force tensiometry; this technique was then used to study the interaction of NG_pos_ with model proteins Fib and IgG. These proteins have been widely used along with BSA for protein corona studies, due to their abundance in the corona of different NPs and to their role in the process of nanomaterial opsonization and immune recognition [89,90]. 

**Figure 3:**
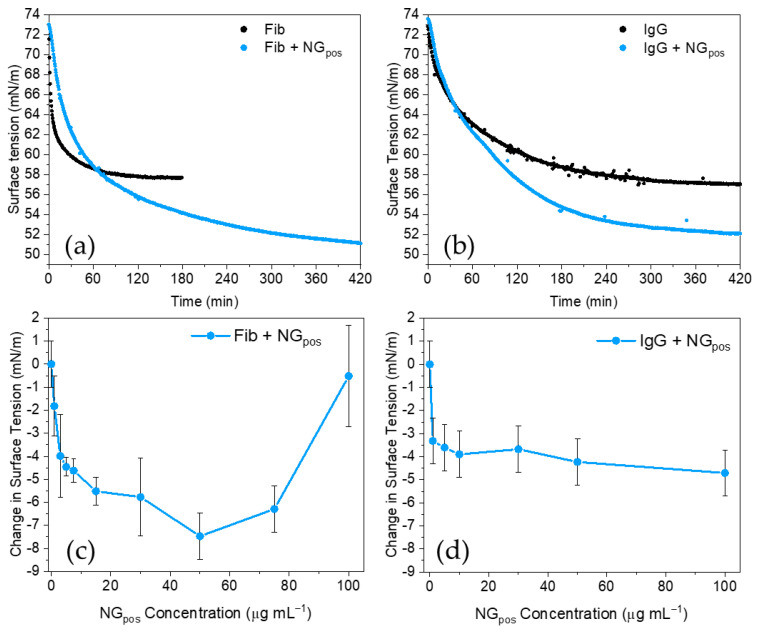
Protein corona studies of NG_pos_ with model proteins Fib and IgG. Dynamic surface tension plots for NG_pos_ (50 μg mL^−1^) with: (**a**) Fib, or (**b**) IgG. Change in equilibrium surface tension of protein samples as a function of the concentration of NG_pos_ are presented for: (**c**) Fib, and (**d**) IgG.

The surface tension profiles of Fib (75 μg mL^−1^) and IgG (100 μg mL^−1^) were first measured in the PBS buffer (Figure 3a,b black dots), with values of surface tension at equilibrium in agreement with previously reported studies (Table 3) [91,92,93]. Equilibration times for Fib and IgG were found to be around 2 and 5 h, respectively, and significantly higher than for BSA. Although this might be attributed mainly to the larger molecular weight of Fib (350 kDa) and IgG (150 kDa) compared to BSA (66 kDa), other factors such as the hydrophilic/hydrophobic amino acid ratio and overall protein rigidity contribute to the individual protein kinetic of adsorption at the interface [78,94,95]. Thus, the different kinetics of adsorption are indicative of the different chemical properties of the model proteins in this study.

The addition of NG_neut_ and NG_neg_ (100 μg mL^−1^) to Fib and IgG led to no significant changes in adsorption kinetics or equilibrium surface tension values, with the exception of mixtures of Fib and NG_neut_ (Table 3, see Figure S10 for complete runs). In this case, a slightly lower surface tension was measured for the NG_neut_-Fib mixture compared to pure Fib (from 58.3 mN m^−1^ to 56.1 mN m^−1^), indicating the formation and adsorption of their complex at the air/water interface. Given the hydrophilic character and neutral charge of NG_neut_, it is reasonable to expect hydrogen bonding to be driving the formation of this complex. Although hydrophilic surfaces are generally associated with low-fouling properties [96,97], the presence of hydrogen donating groups on the surface of NPs has been linked to a stronger affinity towards protein adsorption on both NPs and surfaces [98,99,100]. Moreover, hydrophilic NPs have been observed to have stronger affinity for Fib compared to BSA and IgG [67,101,102], in agreement with our findings. It is important to highlight that no formation of NG_neut_-Fib complexes could be observed by DLS (Figure S11a), thus demonstrating the higher resolution offered by surface tensiometry. The subtle interactions in the bulk, that cannot be observed with traditional methodologies, may still influence the interfacial properties of nanogels, thus impacting their performance as drug delivery systems. 

The addition of NG_pos_ to Fib and IgG solutions significantly affected their surface tension profiles (Figure 3a,b). As found for BSA, the addition of NG_pos_ resulted in a lower surface tension at equilibrium, indicating a formation of complexes between NG_pos_ and proteins in solution and further adsorption at the air/water interface. NG_pos_-Fib featured slower kinetics of adsorption compared to Fib (Figure 3a), indicating that complex formation was associated with an increase in size of the nanogels, as confirmed by DLS (Figure S11), and thus a reduced rate of diffusion to the air/water interface. Interestingly, the addition of IgG to NG_pos_ did not show significant changes in the DLS data (Figure S12), further demonstrating the higher sensitivity of surface tensiometry; formation of particles with a size of around 1000 nm by intensity resulting from NG_pos_-IgG interactions were observed by DLS only when the concentration of the protein was increased to 5 mg mL^−1^ (Figure S13a); however, these larger particles possibly represent a small portion of the sample as observed in the result by number (Figure S13b).

Interestingly, concentration studies revealed a different behaviour for complexes formed with Fib or IgG (Figure 3c,d). On one hand, a complex formation with IgG led to a decrease in surface tension, which reached a plateau after 5 μg mL^−1^ of NG_pos_ was added. On the other hand, the surface tension of NG_pos_-Fib mixtures decreased until a minimum was reached at around 50 μg mL^−1^ of nanogels. Further addition of NG_pos_ led to an increase in the surface tension of the sample, causing an inversion of the trend. As NG_pos_ concentrations rose, a relative longer time of equilibration could be also observed (Figure S5b and Figure S14), indicating that NG_pos_-Fib complexes required more time to adsorb at the interface when more nanogels were added to the solution. This suggests that at low nanogel concentration, NG_pos_-Fib complexes can readily adsorb at the interface, while at higher concentrations larger aggregates with decreased diffusivity have slower kinetics of adsorption to the interface. When the NG_pos_ concentration reached 100 μg mL^−1^, the size of the complexes prevented their adsorption at the interface, causing its depletion. This was confirmed by quantification of the free Fib left in the supernatant, after the complexes were removed from the solution by centrifugation (Figure S15). Data showed depletion of Fib from the solution in the proximity of the peak registered on surface tensiometry. This is evidence that protein corona formation can affect nanogel interfacial behaviour, both in terms of overall interfacial structure but also of kinetic of adsorption at the interface. Moreover, data on BSA, Fib, and IgG show that the type of protein involved in corona formation determines the way nanogels interact with the interface. In fact, interaction of NG_pos_ with BSA and IgG led to a comparable decrease in the sample surface tension (−3.6 mN m^−1^, and −5.5 mN m^−1^ for BSA and IgG, respectively) and negligible effects on the relative rate of adsorption to the interface (Figure S14). Interactions with Fib led to a larger drop in surface tension values of the complex (−8 mN m^−1^ at minimum) together with longer relative adsorption times. These data could indicate a potential role of the protein structure (globular for BSA and IgG, tubular for Fib) in determining their different behaviour at the interface. CD analysis did not provide an evidence of protein denaturation for Fib and IgG in the presence of NG_pos_ (Figure S6b,c). This indicates that, as for BSA, the changes in interfacial behaviour are likely due to variations in the degree of hydrophobicity of the NG-protein complex. 

### 2.5. Protein Corona Analysis of Natural NPs: Extracellular Vesicles

After characterizing the interfacial properties of nanogels, we extended the characterization to other nanomaterials to demonstrate the relevance of the surface tensiometry technique for studying the protein corona. Recently, extracellular vesicles (EVs) have attracted considerable interest due to their natural ability to permeate biological barriers and target specific cells to deliver their cargo [103]. Given the technical challenge associated with the isolation of pure EVs and characterization of their surface-associated molecules [104], their protein corona has only been recently investigated. EVs have been found to associate with different types of biological molecules such as proteins, lipoproteins, or nucleic acids [105,106,107]. Given the limited number of techniques suitable for the study of their corona in situ, surface tensiometry was tested to investigate the protein corona of this nanomaterial. 

To investigate the protein corona of EVs, BSA and Fib were selected as model proteins of globular and tubular structure, respectively. EVs had been isolated from human umbilical cord blood plasma samples and they were purified by ultracentrifugation followed by size exclusion chromatography. These vesicles are negatively charged (−28.9 ± 1.2 mV). As seen in Figure S16, the size of EVs in solution was found to be around 150 nm by DLS as typically reported for EVs in literature [108]. The addition of either BSA or Fib to the EVs solution caused no significant size increase or sign of complex formation. However, protein corona formation by DLS for particles with size > 100 nm may be challenging, as the increase in particle diameter caused by protein adsorption is often smaller than the error associated with the measurements itself. The surface tension of EVs sample in PBS was found to be negligible surface activity over a window of 4 h (Figure S16), making this material suitable for analysis with both BSA and Fib. The surface tensiometry of EV-BSA and EV-Fib solutions are shown in Figure S16c,d. In both cases the addition of EVs was found to have no significant effect on the surface tension values of these two proteins at equilibrium or on their equilibration time. This result indicates that EVs behave differently from the positively charged nanogels, and do not form a protein corona with BSA and Fib. 

## 3. Materials and Methods

### 3.1. Materials

All chemicals were used as received unless otherwise stated. Acrylamide (AM, 99%), *N,N′*-methylene-bisacrylamide (MBA, 99%), 1,2,4,5-tetramethylbenzene (TMB, 98%), 2-ethyl-2-thiopseudourea hydrobromide (98%), and triethylamine (TEA, >99.5%) were purchased from Sigma Aldrich (Gillingham, UK). 2-Acrylamido-2-methyl-1-propanesulfonic acid (AMPS, 98%) was purchased from Alfa Aesar (Heysham, UK). *N*-(3-aminopropyl) methacrylamide chloride (95%) was obtained from Fluorochem. 2,2′-azobisisobutyronitrile (AIBN, 98%) was purchased from Sigma Aldrich and used after recrystallization from methanol. Deuterated acrylamide (d3-AM, 98%) was obtained from Polymer Source (Dorval, Canada). Acryloyl chloride (96% with 400 ppm phenothiazine stabilizer) was purchased from Alfa Aesar (Heysham, UK). Dry DMSO was purchased from Goss Scientific (Crewe, UK), while deuterated DMSO (DMSO-6*d*) employed for NMR conversion studies was obtained from Cambridge Isotope Laboratories. Acetonitrile was obtained from Fisher, while methanol was received from Honeywell. Chloroform was purchased from VWR. The cellulose dialysis membrane (molecular weight cut-off: 3500 Da, width: 34 mm, diameter: 22 mm) was purchased from Medicell International Ltd. (London, UK). Bovine serum albumin (BSA, code A7030, ≥98%), human fibrinogen (Fib, code F3879, 50–70%), and human immunoglobulin G (IgG, code I4506, ≥95%) were obtained from Sigma Aldrich (Gillingham, UK). Polytetrafluoroethylene (PTFE) syringe filters with pore size 0.2 μm and polyethersulfone (PES) syringe filters with pore size 0.2 and 0.45 μm were obtained from Fisher Scientific (Loughborough, UK).

### 3.2. Nanogel Synthesis

Nanogels were synthesized via high dilution radical polymerisation (HDRP) following our previously reported procedure [109]. AM (backbone monomer), MBA (covalent crosslinker), *N*-(3-methacrylamidopropyl) guanidinium chloride (GUA) (positively charged co-monomer), and AMPS (negatively charged co-monomer) were dissolved in an appropriate volume of anhydrous DMSO to yield a total monomer concentration of either 1 or 2% (*w*/*w*) depending on individual formulations. The concentration of crosslinker MBA was fixed at 20 mol%, which is the percentage of moles of crosslinker based on the total moles of monomers in the formulation.

The initiator, AIBN, was added to the monomer solution in concentrations of 1 or 2 mol% of total moles of double bonds present in the mixture. 

The solution of monomers and initiator was then transferred in a Wheaton™ bottle, the vessel was sealed and purged with N_2_ for 15 min before being heated at 70 °C for 24 h. The resulting nanogel’s clear suspension in DMSO was purified via dialysis (MWCO 3500 Da, diameter 22 mm) against deionised water for 3 days changing water thrice a day. The purified nanogel solution in water was then freeze-dried (LTE Scientific Lyotrap, Greenfield, UK), yielding a soft white powder that was stored at room temperature. Chemical yields were determined by weighing the dry polymer. The same protocol was used when partially deuterated nanogel were prepared for the NR experiments.

The nanogels were reconstituted to form a stable colloidal solution by dispersing the required amount of dry polymer in the aqueous solution (depending on the requirement of the experiment), followed by 10 min sonication of the solution at room temperature and filtration of the resulting clear solution through a 0.2 µm PTFE.

### 3.3. Quantification of Monomer Conversions by ^1^H-NMR Spectroscopy

Aliquots (250 μL) of the polymerisation mixture were drawn immediately after monomer complete solubilization (time zero, t_0h_) and after the reaction was quenched (time 24 h, t_24h_) using a microsyringe. The aliquots were then mixed with 250 μL of 1,2,4,5-tetramethylbenzene stock solution (8 mg/mL) in DMSO-d6 as the internal standard and transferred into an NMR tube. ^1^H-NMR spectra were recorded in the solvent suppression mode at 298 K using a Brucker HD400, or Bruker AVIII400 spectrometer (400 MHz). Spectra were processed with Mestrenova software (version 6.0.2-5475). The ^1^H-NMR spectra acquired were phased, baseline corrected, and integrated identically. The concentration of monomers and crosslinker in the initial and final polymerization solutions were determined by comparing the intensities of monomer peaks at 6.07 ppm for AM, 5.63 ppm for MBA, 5.51 ppm for AMPS, and at 5.67 ppm for GUA against the intensities of peaks of the internal standard at 6.88 ppm (1,2,4,5-tetramethylbenzene). Chemical yields of nanogels were estimated by subtracting the amount of polymerisation mixture withdrawn during the NMR conversion study.

### 3.4. Dynamic Light Scattering 

Hydrodynamic diameter (D_h_) measurements were obtained by DLS at 25 °C using a Zetasizer Nano Ultra operated with the software ZS Xplorer (version 1.5.0.163) (Malvern Instruments Ltd., Malvern, UK). Nanogels stock colloidal solutions (1 mg mL^−1^) were obtained by dissolving the dry nanogel powder in PBS (10 mM, pH 7.4), followed by sonication for 10 min and filtration of the resulting clear solution through a 0.2 µm PTFE. This nanogel solution was then diluted to a concentration of 0.1 mg mL^−1^ with filtered PBS (0.2 µm PTFE) prior to analysis. 

For protein corona analysis, proteins stock solutions were obtained by dissolving powders in PBS without agitation. BSA and IgG clear solutions were then filtered through a 0.2 µm PES filter prior to the analysis. Due to its larger size, Fib was filtered with a 0.45 µm PES filter instead to avoid sample loss. Protein solutions were then diluted with either filtered PBS (0.2 µm PTFE) or nanogels solution to achieve the desired concentration for protein corona studies.

To avoid contamination from airborne dust, all samples were filtered under a fume hood and cuvettes were flushed with air immediately before samples were added. All measurements were carried out in triplicate using backscatter (173°) angle mode, allowing 10 min for sample temperature to equilibrate prior to each measurement. 

### 3.5. Characterisation of Nanogels via Z-Potential Analysis

Z-potential analysis of nanogels was conducted at 25 °C using a Zetasizer Nano Ultra operated with the software ZS Xplorer (version 1.5.0.163) (Malvern Instruments Ltd., Malvern, UK). Nanogels stock colloidal solutions (1 mg mL^−1^) were obtained by dissolving the dry nanogel powder in PB (10 mM, pH 7.4), followed by sonication for 10 min and filtration of the resulting clear solution through a 0.2 µm PTFE into a disposable folded capillary cell (1080, Malvern Instruments Ltd., Malvern, UK). 

### 3.6. Synthesis of GUA

GUA was synthesized according to a modified literature procedure [110]. Briefly, *N*-(3-aminopropyl) methacrylamide chloride (999 mg, 5.60 mmol) and 2-ethyl-2-thiopseudourea hydrobromide (980 mg, 5.32 mmol) were added to a 25 mL bottom round flask together with 10 mL of acetonitrile (AcCN) and 0.5 mL of deionised water. To this solution, triethylamine (1560 μL, 11.2 mmol) was then added. The reaction was stirred at room temperature for 16 h, after which, the solvent was removed under reduced pressure. The resulting colorless oil was purified via column chromatography (eluent: 5% MeOH in CH_3_Cl followed by 25% MeOH in CH_3_Cl) over alumina (50–200 μm 60 Å, Rf = 0.32). The product was obtained as a clear oil, 1.015 g (87%). ^1^H-NMR (400 MHz, DMSO) δ 8.01 (t, J = 5.4 Hz, 1H), 7.68 (t, J = 5.7 Hz, 1H), 7.6–6.7 (br, 4H), 5.68 (dd, J = 1.4, 1.0 Hz, 1H), 5.33 (p, J = 1.5 Hz, 1H), 3.23–3.04 (m, 4H), 1.86 (dd, J = 1.5, 0.9 Hz, 1H), 1.78–1.56 (m, 2H).

### 3.7. Static Surface Tensiometry of Nanogels

The surface tension of pure nanogels in PBS (10 mM, pH 7.4) was evaluated with a Kruss K9 (Kruss Scientific, Hamburg, Germany) force tensiometer equipped with a glass through and a Wilhelmy plate (PL01) as probe. All samples were measured at 25 °C. Nanogels stock colloidal solutions were prepared in the same way as reported in Section 3.4. Nanogel solutions were then diluted with filtered PBS (0.2 µm PTFE) to obtain samples with appropriate concentrations for analysis. Surface tension measurements were collected at 0.5, 1, 3, 5, 8 and 24 h from the start of the experiment for each nanogel sample. At each time point, 5 consecutive measurements were performed, and the average was used to report the surface tension value. 

### 3.8. Protein Corona Studies by Dynamic Surface Tensiometry 

The kinetics of adsorption at the air/water interface of proteins and nanogel-proteins mixtures were analysed using a Kruss K100 Force Tensiometer (Kruss Scientific, Hamburg, Germany) equipped with a PTFE sample trough (SV01, volume 6.4 mL) and a Wilhelmy plate (PL02). All samples were measured at 25 °C. Nanogels and proteins stock solutions were prepared as per Section 3.4. Then, the protein stock solutions were diluted with either filtered PBS (0.2 µm PTFE) or nanogels solution to obtain samples with the desired concentration for protein corona studies. As soon as samples were prepared, they were loaded on a 6.4 mL PTFE trough and the measurement was immediately started. Surface tension measurements were collected every 30 s until a plateau was reached. The final surface tension values were taken as average of the last 10 measurements after reaching a plateau. Each measurement was repeated at least twice. Prior to every experiment, pure PBS was measured to confirm no contamination of the set-up (surface tension of pure PBS 72 ± 0.5 mN m^−1^). 

### 3.9. Protein Corona Studies by Neutron Reflectometry

NR experiments were carried out on the FIGARO reflectometer (10.5291/ILL-DATA.9-12-588 and 10.5291/ILL-DATA.9-13-838) at the Institut Laue-Langevin (ILL), Grenoble, France. The NR profiles were measured at two incident angles (θ = 0.624° and 3.78°) to provide a wide Q-range, hence the highest sensitivity to the interfacial structure. The sample was illuminated at a resolution δQ/Q ≈ 8%. The water sub-phase was D_2_O, contrast-matched to air (CMAir) or contrast-matched to BSA (CMBSA). To enhance the contrast during NR experiments, partial deuterated NGpos nanogels, made of 60%AM-d3 + 20% MBA + 20% GUA, were used for CMAir and CMBSA subphases. In the case of D_2_O contrast, the normal NG_pos_ (60% AM + 20% MBA + 20% GUA) sample was used. Scattering length and scattering length density (SLD) values of BSA and nanogels used in this work are shown Table S3.

NR data were analysed in refnx software (version 0.1.30) using the Markov chain Monte Carlo (MCMC) fit algorithm. The analysis of NR data includes generating model parameters such as SLD thickness, roughness, and number of layers, calculating the reflectivity, and comparing the result to the data obtained from the experiments. This process was repeated until the differences between the global fit and the experimental data in different isotopic contrasts had reached a minimum. The optimal fitted parameters were combined to give the scattering length density profile of the sample, which represents the variation in composition perpendicular to the interface. The nature of model fitting of NR data necessitates finding the simplest model, i.e., the model with the minimum number of fitting parameters.

When resolving the volume fraction and adsorbed amount of materials in each layer in each layer in the NR data recorded at the air/water interface, we assumed that there were no air bubbles inside the interfacial layers. As a result

$$\underset{i}{\sum }(SLD_{i}*VF_{i})=SLD_{fit}$$

where *SLD_i_* and *VF_i_* stand for the SLD and volume fraction of component *i* (could be nanogel, BSA, or water) whereas *SLD_fit_* is the SLD value from the fit. As two or three different isotopic contrasts were used, it was possible to calculate the volume fraction of water, BSA, and nanogels in each layer. Theoretically speaking, the sum of the volume fractions of all components should equal 1, which is an extra constraint to resolve the volume fraction profiles.

The adsorbed amount of nanogel and BSA at the interface can be calculated from the volume fraction values:

Γ=VF∗ρ∗d

where *ρ* is the density of dry BSA or nanogels and *d* is the fitted layer thickness. 

Model fitting is the most used method to analyze Neutron Reflectivity data. The interface region is divided into a finite number of layers, each characterized by a certain layer thickness, Scattering Length Density (SLD), and roughness. The scattering length density can be written as Nb = $\sum_{i}N_{i}b_{i}$ with N_i_ the number density, b_i_ the coherent scattering length. The multiple Nb is known as the SLD. Since Nb is approximately linearly related to the volume fraction composition Nb = $\sum_{i}\emptyset_{i}{\mathrm{Nb}}_{i}$ ($\emptyset_{i}$ is the volume fraction and ${\mathrm{Nb}}_{i}$ is the scattering length density of species i) a layer model with discrete strata representing the interfacial regions with different chemical compositions is then constructed and the reflectivity from such the model is calculated using the classical optical matrix method. The calculated reflectivity profile using an iterative model fitting is carried out and the layer thickness, SLD and roughness are optimised. The fitted profile is then compared to the experimental data and the goodness of the fit is evaluated in terms of χ^2^.

### 3.10. Quantification of Unbound Fibrinogen in Protein Corona Study by UV-Vis Spectroscopy

The interaction of positively charged nanogels (NG_pos_) with Fib were evaluated by UV-Vis spectroscopy. NG_pos_ and Fib were reconstituted in PBS (10 mM, pH 7.4) as described in Section 3.4. Then, Fib and NG_pos_ solutions were mixed to reach a final volume of 1 mL (Fib concentration 0.75 mg mL^−1^, NG_pos_ concentration ranging between 0.01 and 1 mg mL^−1^). The formed solutions were then transferred in Eppendorf tubes and centrifuged at 16,873× *g* for 30 min. Pure NG_pos_ or Fib led to no pellet to be formed. Then, concentration of free Fib was assessed by measuring the absorbance of the supernatant at 280 nm against a calibration curve. 

### 3.11. Circular Dichroism

CD spectra were obtained with a Chirascan spectrometer (Applied Photophysics, Ltd., Leatherhead, UK) using a 1 mm quartz cell (110-4-40 Hellma Analytics, Baden-Wurttemberg, Germany). The CD spectra of nanogels, proteins, and their mixtures in PBS (10 mM, pH 7.4) were recorded from 190 to 260 nm (bandwidth 1 nm, time-per-point 1 s). Data analysis was performed with Chirascan Pro-Data Viewer software (version 4.5.1848.0). Typically, three scans were acquired and averaged. The resulting average spectrum was subtracted from the contribution of buffer (and nanogels when appropriate) and all spectra were zeroed at 260 nm. 

### 3.12. EV Isolation from hUCB-Plasma and Protein Corona Studies

Human umbilical cord blood (hUCB) samples were obtained upon signed informed consent, in compliance with Portuguese legislation. The collection was approved by the ethical committee of the Centro Hospitalar e Universitário de Coimbra, Portugal (HUC-01-11). Briefly, blood plasma was isolated by density gradient separation (Lymphoprep—StemCell Technologies SARL, Grenoble, France). For EV isolation, blood plasma was centrifuged at 2000× *g* for 20 min at 4 °C to pellet cell debris. Using an Optima™ XPN 100K ultracentrifuge with a swinging bucket rotor SW 32 Ti (Beckham Coulter, Brea, CA, USA), the supernatant was centrifuged twice at 10,000× *g* for 30 min at 4 °C in order to pellet larger vesicles. EVs were then centrifuged at 100,000× *g* centrifugation for 120 min at 4 °C. Pelleted EVs were washed with cold PBS and run through commercially available SEC columns (qEV columns, Izon Science, Christchurch, New Zealand) in order to separate EVs from soluble proteins. The EVs fraction was collected and centrifuged one last time at 100,000× *g*, resuspended in 150–200 μL of cold sterile PBS, and stored at −80 °C.

For protein corona studies, EVs were slowly thawed on ice prior to each experiment. To study interactions of EVs with proteins using DLS, EVs (final concentration ~0.08 nM) were mixed with either BSA or Fib (final concentrations 3 mg mL^−1^ and 2.25 mg mL^−1^, respectively) solutions prepared as described in Section 3.4. Samples were equally diluted to adapt the procedure to surface tensiometry measurements (final concentration sEVs 0.002 nM, BSA 100 mg mL^−1^, and Fib 0.075 mg mL^−1^). Pure sEVs were also measured as control by diluting the stock with PBS. 

## 4. Conclusions

The formation of NPs-proteins complexes and the study of their behavior with biological interfaces, such as membranes and cell surfaces, are an essential priority for any application of nanomaterials requiring interactions with biosystems, such as in the case of drug delivery systems and sensors. The evaluation of these interactions in solutions, using in situ analytical techniques, allows to understand the behavior of the systems, without any disruptive isolation step. We have focused on the characterization of the complex formation between acrylamide-based nanogels, neutral and charged, with a range of proteins, such as BSA, Fib, and IgG, which are commonly found in the protein corona of NPs. 

Surface force tensiometry was used, complementing the more traditional and widely used DLS, to demonstrate that complex formation occurs between positively charged nanogels and BSA and that the interfacial behavior of the proteins and NPs is changed. The structure of the BSA-NG_pos_ was resolved by NR experiments on ångström length scale at the air/water interface, with air being the hydrophobic component. The data allowed us to identify that the interfacial structure of the complex is organized in three regions, with a top layer densely packed, followed by a highly solvated middle layer, followed by a lower layer with more polymeric chains diffusing to the bulk phase. The interesting feature is that experiments with varying concentrations of BSA, ranging from 0.02 to 0.5 mg ml^−1^ showed that the SLD value of the middle layer increased gradually towards the aqueous phase, while the other layers remained similar. This confirmed that increasing concentrations of BSA led to more complex to form in the bulk, which resulted in more material being adsorbed at the interface extending towards the aqueous subphase.

Surface tensiometry and DLS were also used to evaluate the complex formation of nanogels with Fib and IgG, demonstrating the pivotal role of electrostatic interactions. These complexes showed the ability to adsorb at weakly hydrophobic interface (air/water) with interfacial properties that differed from that of pure proteins and nanogels. We found that the type of protein involved in the formation of the corona directly impacted the interfacial properties of the complexes both in terms of kinetics of adsorption and structure at the interface, demonstrating the importance of protein structure. The data interestingly show a different behavior of the complexes at the interface, as a result of changes in concentrations. The consistency of behavior of BSA and IgG, both globular proteins, compared to Fib, a tubular protein, prompted us to hypothesize that protein structure and shape may lead to different interfacial behaviors. The combination of techniques used for this study allowed us to demonstrate that surface tensiometry, a simple and easily accessible technique, can provide important information on the in situ behavior of NPs-protein complexes.

This work provides important advances in understanding the role of protein interactions in altering the interfacial behavior of nanogels, which can contribute to the development of NPs for drug delivery applications.

## Figures and Tables

**Table 1:** Chemical composition, monomer conversions and characterization of nanogels with neutral (NG_neut_), positive (NG_pos_), or negative (NG_neg_) surface charge. Monomer conversion was estimated via ^1^H-NMR.

Nanogel	Composition							Monomer Conversion ^a^				Characterization		
	AM	MBA	GUA	AMPS	AIBN	C_M_^b^	Yield	AM	MBA	Co-Monomer	Total	D_h_^c^	PDI	Z-Pot ^d^
	mol%					%	%					nm	-	mV
NG_neut_	80	20	-	-	1	1	74	84	98	-	87	7.6 ± 1.0	0.31	-
NG_pos_	60	20	20	-	2	2	75	93	99	98	95	9.7 ± 0.8	0.46	9.1 ± 1.2
NG_neg_	60	20	-	20	2	1	71	91	97	76	89	5.1 ± 0.4	0.32	−14.5 ± 3.2

Polymerization conditions in all cases: T = 70 °C, 24 h in DMSO. ^a^ Monomer conversion calculated by ^1^H NMR analysis of the initial and final polymerisation mixtures. ^b^ C_M_ is total monomer concentration. ^c^ Hydrodynamic diameter (D_h_) by number distribution and PDI of nanogels (0.1 mg mL^−1^) were measured by dynamic light scattering (backscatter) in PBS (10 mM, pH 7.4). ^d^ Z-potential of nanogels (1 mg mL^−1^) was measured in PB buffer (10 mM, pH 7.4).

**Table 2:** Volume fraction (VF) of BSA-NG_pos_ mixtures at air/water interface.

Layer	VF of BSA	VF of NG_pos_	VF of Water
upper	~40%	~4%	~63%
middle	~2%	~15%	~80%
lower	0%	6%	94%

**Table 3:** Equilibrium surface tension values obtained for Fib, IgG, and their mixtures with neutral (NG_neut_), positively charged (NG_pos_), or negatively charged (NG_neg_) nanogels. Samples were prepared in PBS (pH 7.4) and analysed at 25 °C.

Nanogels	NG Conc. (μg mL^−1^)	Fib Conc. (μg mL^−1^)	IgG Conc. (μg mL^−1^)	Equilibrium Surface Tension (mN m^−1^)	
				Fib	IgG
-	-	-	-	58.3 ± 1.0	60.2 ± 1.1
NG_neut_	100	75	100	56.1 ± 0.2	60.2 ± 1.6
NG_pos_	50			50.3 ± 1.0	54.7 ± 2.1
NG_neg_	100			57.0 ± 1.1	58.6 ± 1.5

## Data Availability

The data presented in this study are available on request from the corresponding author.

## Supplementary Materials

The following supporting information can be downloaded at: https://www.mdpi.com/article/10.3390/ijms24032810/s1.

## References

[1] Huang P., Chaney E.J., Aksamitiene E., Barkalifa R., Spillman D.R., Borgan B.J., Boppart S.A. (2021). Biomechanical Sensing of in Vivo Magnetic Nanoparticle Hyperthermia-Treated Melanoma Using Magnetomotive Optical Coherence Elastography. Theranostics.

[2] Gómez-López P., Puente-Santiago A., Castro-Beltrán A., Santos do Nascimento L.A., Balu A.M., Luque R., Alvarado-Beltrán C.G. (2020). Nanomaterials and Catalysis for Green Chemistry. Curr. Opin. Green Sustain. Chem..

[3] Mitchell M.J., Billingsley M.M., Haley R.M., Langer R., Wechsler M.E., Peppas N.A. (2021). Engineering Precision Nanoparticles for Drug Delivery. Nat. Rev. Drug Discov..

[4] Patil Y., Sadhukha T., Ma L., Panyam J. (2009). Nanoparticle-Mediated Simultaneous and Targeted Delivery of Paclitaxel and Tariquidar Overcomes Tumor Drug Resistance. J. Control. Release.

[5] Blanco E., Shen H., Ferrari M. (2015). Principles of Nanoparticle Design for Overcoming Biological Barriers to Drug Delivery. Nat. Biotechnol..

[6] Koo O.M., Rubinstein I., Onyuksel H. (2005). Role of Nanotechnology in Targeted Drug Delivery and Imaging: A Concise Review. Nanomed. Nanotechnol. Biol. Med..

[7] Najahi-Missaoui W., Arnold R.D., Cummings B.S. (2021). Safe Nanoparticles: Are We There Yet?. Int. J. Mol. Sci..

[8] Cedervall T., Lynch I., Foy M., Berggård T., Donnelly S.C., Cagney G., Linse S., Dawson K.A. (2007). Detailed Identification of Plasma Proteins Adsorbed on Copolymer. Angew. Chem. Int. Ed..

[9] Mahmoudi M., Lynch I., Ejtehadi M.R., Monopoli M.P., Bombelli F.B., Laurent S. (2011). Protein—Nanoparticle Interactions: Opportunities and Challenges. Chem. Rev..

[10] Zhang X., Pandiakumar A.K., Hamers R.J., Murphy C.J. (2018). Quantification of Lipid Corona Formation on Colloidal Nanoparticles from Lipid Vesicles. Anal. Chem..

[11] Lima T., Bernfur K., Vilanova M., Cedervall T. (2020). Understanding the Lipid and Protein Corona Formation on Different Sized Polymeric Nanoparticles. Sci. Rep..

[12] Zeng Z., Patel J., Lee S., Mccallum M., Tyagi A., Yan M., Shea K.J. (2012). Synthetic Polymer Nanoparticle − Polysaccharide Interactions: A Systemtic Study. J. Am. Chem. Soc..

[13] Li K., Zhao X., Hammer B.K., Du S., Chen Y., Engineering E., States U., States U. (2013). Nanoparticles Inhibit DNA Replication by Binding to DNA: Modeling and Experimental Validation. ACS Nano.

[14] Mosquera J., Garciá I., Henriksen-Lacey M., Martínez-Calvo M., Dhanjani M., Mascarenãs J.L., Liz-Marzán L.M. (2020). Reversible Control of Protein Corona Formation on Gold Nanoparticles Using Host-Guest Interactions. ACS Nano.

[15] Bertrand N., Grenier P., Mahmoudi M., Lima E.M., Appel E.A., Dormont F., Lim J.M., Karnik R., Langer R., Farokhzad O.C. (2017). Mechanistic Understanding of in Vivo Protein Corona Formation on Polymeric Nanoparticles and Impact on Pharmacokinetics. Nat. Commun..

[16] Kong H., Xia K., Ren N., Cui Y., Liu R., Li Q., Lv M., Shi J., Yan Q., Cui Z. (2018). Serum Protein Corona-Responsive Autophagy Tuning in Cells. Nanoscale.

[17] Musyanovych A., Fetz V., Tenzer S., Docter D., Hecht R., Schlenk F., Fischer D., Kiouptsi K., Reinhardt C., Maskos M. (2013). Rapid Formation of Plasma Protein Corona Critically Affects Nanoparticle Pathophysiology. Nat. Nanotechnol..

[18] Salvati A., Pitek A.S., Monopoli M.P., Prapainop K., Bombelli F.B., Hristov D.R., Kelly P.M., Åberg C., Mahon E., Dawson K.A. (2013). Transferrin-Functionalized Nanoparticles Lose Their Targeting Capabilities When a Biomolecule Corona Adsorbs on the Surface. Nat. Nanotechnol..

[19] Caracciolo G., Cardarelli F., Pozzi D., Salomone F., Maccari G., Bardi G., Capriotti A.L., Cavaliere C., Papi M., Laganà A. (2013). Selective Targeting Capability Acquired with a Protein Corona Adsorbed on the Surface of 1,2-Dioleoyl-3-Trimethylammonium Propane/Dna Nanoparticles. ACS Appl. Mater. Interfaces.

[20] Smolková B., MacCulloch T., Rockwood T.F., Liu M., Henry S.J.W., Frtús A., Uzhytchak M., Lunova M., Hof M., Jurkiewicz P. (2021). Protein Corona Inhibits Endosomal Escape of Functionalized DNA Nanostructures in Living Cells. ACS Appl. Mater. Interfaces.

[21] Champion J.A., Pustulka S.M., Ling K., Pish S.L. (2020). Protein Nanoparticle Charge and Hydrophobicity Govern Protein Corona and Macrophage Uptake. ACS Appl. Mater. Interfaces.

[22] Voronovic E., Skripka A., Jarockyte G., Ger M., Kuciauskas D., Kaupinis A., Valius M., Rotomskis R., Vetrone F., Karabanovas V. (2021). Uptake of Upconverting Nanoparticles by Breast Cancer Cells: Surface Coating versus the Protein Corona. ACS Appl. Mater. Interfaces.

[23] Kim K., Kim J., Back J.H., Lee J.E., Ahn D. (2022). Cholesterol-Mediated Seeding of Protein Corona on DNA Nanostructures for Targeted Delivery of Oligonucleotide Therapeutics to Treat Liver Fibrosis. ACS Nano.

[24] Lou Z., Han H., Mao D., Jiang Y., Song J. (2018). Qualitative and Quantitative Detection of PrPSc Based on the Controlled Release Property of Magnetic Microspheres Using Surface Plasmon Resonance (SPR). Nanomaterials.

[25] Weber C., Morsbach S., Landfester K. (2019). Possibilities and Limitations of Different Separation Techniques for the Analysis of the Protein Corona. Angew. Chem. Int. Ed..

[26] Böhmert L., Voß L., Stock V., Braeuning A., Lampen A., Sieg H. (2020). Isolation Methods for Particle Protein Corona Complexes from Protein-Rich Matrices. Nanoscale Adv..

[27] Pederzoli F., Tosi G., Vandelli M.A., Belletti D., Forni F., Ruozi B. (2017). Protein Corona and Nanoparticles: How Can We Investigate On?. WIREs Nanomed. Nanobiotechnol..

[28] Winzen S., Schoettler S., Baier G., Rosenauer C., Mailaender V., Landfester K., Mohr K. (2015). Complementary Analysis of the Hard and Soft Protein Corona: Sample Preparation Critically Effects Corona Composition. Nanoscale.

[29] Casals E., Pfaller T., Duschl A., Oostingh G.J., Puntes V. (2010). Time Evolution of the Nanoparticle Protein Corona. ACS Nano.

[30] Mohammad-Beigi H., Hayashi Y., Zeuthen C.M., Eskandari H., Scavenius C., Juul-Madsen K., Vorup-Jensen T., Enghild J.J., Sutherland D.S. (2020). Mapping and Identification of Soft Corona Proteins at Nanoparticles and Their Impact on Cellular Association. Nat. Commun..

[31] Di Silvio D., Maccarini M., Parker R., Mackie A., Fragneto G., Baldelli Bombelli F. (2017). The Effect of the Protein Corona on the Interaction between Nanoparticles and Lipid Bilayers. J. Colloid Interface Sci..

[32] Sanchez-Guzman D., Giraudon-Colas G., Marichal L., Boulard Y., Wien F., Degrouard J., Baeza-Squiban A., Pin S., Renault J.P., Devineau S. (2020). In Situ Analysis of Weakly Bound Proteins Reveals Molecular Basis of Soft Corona Formation. ACS Nano.

[33] Ge F., Xue J., Wang Z., Xiong B., He Y. (2019). Real-Time Observation of Dynamic Heterogeneity of Gold Nanorods on Plasma Membrane with Darkfield Microscopy. Sci. China Chem..

[34] Prozeller D., Morsbach S., Landfester K. (2019). Isothermal Titration Calorimetry as a Complementary Method for Investigating Nanoparticle-Protein Interactions. Nanoscale.

[35] Shang L., Nienhaus G.U. (2017). In Situ Characterization of Protein Adsorption onto Nanoparticles by Fluorescence Correlation Spectroscopy. Acc. Chem. Res..

[36] Lacerda S.H.D.P., Park J.J., Meuse C., Pristinski D., Becker M.L., Karim A., Douglas J.F. (2010). Interaction of Gold Nanoparticles with Common Human Blood Proteins. ACS Nano.

[37] Carrillo-Carrion C., Carril M., Parak W.J. (2017). Techniques for the Experimental Investigation of the Protein Corona. Curr. Opin. Biotechnol..

[38] Sheibani S., Basu K., Farnudi A., Ashkarran A., Ichikawa M., Presley J.F., Bui K.H., Ejtehadi M.R., Vali H., Mahmoudi M. (2021). Nanoscale Characterization of the Biomolecular Corona by Cryo-Electron Microscopy, Cryo-Electron Tomography, and Image Simulation. Nat. Commun..

[39] Kokkinopoulou M., Simon J., Landfester K., Mailänder V., Lieberwirth I. (2017). Visualization of the Protein Corona: Towards a Biomolecular Understanding of Nanoparticle-Cell-Interactions. Nanoscale.

[40] Feiner-gracia N., Beck M., Pujals S., Tosi S., Mandal T., Buske C., Linden M., Albertazzi L. (2017). Super-Resolution Microscopy Unveils Dynamic Heterogeneities in Nanoparticle Protein Corona. Small.

[41] Wang Y., Rodriguez P.E.D.S., Woythe L., Samuel S., Samitier J., Zijlstra P., Albertazzi L. (2022). Multicolor Super-Resolution Microscopy of Protein Corona on Single Nanoparticles. ACS Appl. Mater. Interfaces.

[42] Sebastiani F., Arteta M.Y., Lerche M., Porcar L., Lang C., Bragg R.A., Elmore C.S., Krishnamurthy V.R., Russell R.A., Darwish T. (2021). Apolipoprotein E Binding Drives Structural and Compositional Rearrangement of MRNA- Containing Lipid Nanoparticles. ACS Nano.

[43] Fleury J., Werner M., Le X., Baulin V.A. (2021). Protein Corona Modulates Interaction of Spiky Nanoparticles with Lipid Bilayers. J. Colloid Interface Sci..

[44] Lin X., Pan Q., He Y. (2019). In Situ Detection of Protein Corona on Single Particle by Rotational Diffusivity. Nanoscale.

[45] Shourni S., Javadi A., Hosseinpour N., Bahramian A., Raoufi M. (2022). Characterization of Protein Corona Formation on Nanoparticles via the Analysis of Dynamic Interfacial Properties: Bovine Serum Albumin—Silica Particle Interaction. Colloids Surf. A Physicochem. Eng. Asp..

[46] Dawson K.A., Yan Y. (2021). Current Understanding of Biological Identity at the Nanoscale and Future Prospects. Nat. Nanotechnol..

[47] Molina M., Asadian-birjand M., Balach J., Bergueiro J. (2015). Stimuli-Responsive Nanogel Composites and Their Application in Nanomedicine. Chem. Soc. Rev..

[48] Soni K.S., Desale S.S., Bronich T.K. (2016). Nanogels: An Overview of Properties, Biomedical Applications and Obstacles to Clinical Translation. J. Control. Release.

[49] Chacko R.T., Ventura J., Zhuang J., Thayumanavan S. (2013). Polymer Nanogels: A Versatile Nanoscopic Drug Delivery Platform. Adv. Drug Deliv. Rev..

[50] Pellizzoni E., Tommasini M., Marangon E., Rizzolio F., Saito G., Benedetti F., Toffoli G., Resmini M., Berti F. (2016). Biosensors and Bioelectronics Fluorescent Molecularly Imprinted Nanogels for the Detection of Anticancer Drugs in Human Plasma. Biosens. Bioelectron..

[51] Anastasiadi R.M., Berti F., Colomban S., Tavagnacco C., Navarini L., Resmini M. (2021). Simultaneous Quantification of Antioxidants Paraxanthine and Caffeine in Human Saliva by Electrochemical Sensing for CYP1A2 Phenotyping. Antioxidants.

[52] Papadimitriou S.A., Robin M.P., Ceric D., O’Reilly R.K., Marino S., Resmini M. (2016). Fluorescent Polymeric Nanovehicles for Neural Stem Cell Modulation. Nanoscale.

[53] Salinas Y., Castilla A.M., Resmini M. (2018). An L-Proline Based Thermoresponsive and PH- Switchable Nanogel as a Drug Delivery Vehicle. Polym. Chem..

[54] Zielińska K., Sun H., Campbell R.A., Zarbakhsh A., Resmini M. (2016). Smart Nanogels at the Air/Water Interface: Structural Studies by Neutron Reflectivity. Nanoscale.

[55] Sun H., Resmini M., Zarbakhsh A. (2018). Interaction of Thermal Responsive NIPAM Nanogels with Model Lipid Monolayers at the Air-Water Interface. J. Colloid Interface Sci..

[56] Sun H., Zielinska K., Resmini M., Zarbakhsh A. (2019). Interactions of NIPAM Nanogels with Model Lipid Multi-Bilayers: A Neutron Reflectivity Study. J. Colloid Interface Sci..

[57] Lundqvist M., Stigler J., Elia G., Lynch I., Cedervall T., Dawson K.A. (2008). Nanoparticle Size and Surface Properties Determine the Protein Corona with Possible Implications for Biological Impacts. Proc. Natl. Acad. Sci. USA.

[58] Yang K., Mesquita B., Horvatovich P., Salvati A. (2020). Tuning Liposome Composition to Modulate Corona Formation in Human Serum and Cellular Uptake. Acta Biomater..

[59] Gossmann R., Fahrländer E., Hummel M., Mulac D., Brockmeyer J., Langer K. (2015). Comparative Examination of Adsorption of Serum Proteins on HSA- and PLGA-Based Nanoparticles Using SDS-PAGE and LC-MS. Eur. J. Pharm. Biopharm..

[60] García-Álvarez R., Hadjidemetriou M., Sánchez-Iglesias A., Liz-Marzán L.M., Kostarelos K. (2018). In Vivo Formation of Protein Corona on Gold Nanoparticles. the Effect of Their Size and Shape. Nanoscale.

[61] Diederichs J.E. (1996). Plasma Protein Adsorption Patterns on Liposomes: Establishment of Analytical Procedure. Electrophoresis.

[62] Monopoli M.P., Walczyk D., Campbell A., Elia G., Lynch I., Bombelli F.B., Dawson K.A. (2011). Physical—Chemical Aspects of Protein Corona: Relevance to in Vitro and in Vivo Biological Impacts of Nanoparticles. J. Am. Chem. Soc..

[63] Tenzer S., Docter D., Rosfa S., Wlodarski A., Kuharev J., Rekik A., Knauer S.K., Bantz C., Nawroth T., Bier C. (2011). Nanoparticle Size Is a Critical Physicochemical Determinant of the Human Blood Plasma Corona: A Comprehensive Quantitative Proteomic Analysis. ACS Nano.

[64] Walkey C.D., Olsen J.B., Song F., Liu R., Guo H., Olsen D.W.H., Cohen Y., Emili A., Chan W.C.W. (2014). Protein Corona Fingerprinting Predicts the Cellular Interaction of Gold and Silver Nanoparticles. ACS Nano.

[65] Maiorano G., Sabella S., Sorce B., Brunetti V., Malvindi M.A., Cingolani R., Pompa P.P. (2010). Effects of Cell Culture Media on the Dynamic Formation of Protein-Nanoparticle Complexes and Influence on the Cellular Response. ACS Nano.

[66] Graham N.B., Cameron A. (1998). Nanogels and Microgels: The New Polymeric Materials Playground. Pure Appl. Chem..

[67] Cedervall T., Lynch I., Lindman S., Berggård T., Thulin E., Nilsson H., Dawson K.A., Linse S. (2007). Understanding the Nanoparticle-Protein Corona Using Methods to Quntify Exchange Rates and Affinities of Proteins for Nanoparticles. Proc. Natl. Acad. Sci. USA.

[68] Merlot A.M., Kalinowski D.S., Richardson D.R. (2014). Unraveling the Mysteries of Serum Albumin-More than Just a Serum Protein. Front. Physiol..

[69] Li Y., Yang G., Mei Z. (2012). Spectroscopic and Dynamic Light Scattering Studies of the Interaction between Pterodontic Acid and Bovine Serum Albumin. Acta Pharm. Sin. B.

[70] Yonamine Y., Yoshimatsu K., Lee S.H., Hoshino Y., Okahata Y., Shea K.J. (2013). Polymer Nanoparticle-Protein Interface. Evaluation of the Contribution of Positively Charged Functional Groups to Protein Affinity. ACS Appl. Mater. Interfaces.

[71] Culver H.R., Sharma I., Wechsler M.E., Anslyn E.V., Peppas N.A. (2017). Charged Poly(N -Isopropylacrylamide) Nanogels for Use as Differential Protein Receptors in a Turbidimetric Sensor Array. Analyst.

[72] Pino P., Pelaz B., Zhang Q., Ulrich G., Parak W.J. (2014). Protein Corona Formation around Nanoparticles—From the Past to the Future. Mater. Horiz..

[73] McClellan S.J., Franses E.I. (2003). Effect of Concentration and Denaturation on Adsorption and Surface Tension of Bovine Serum Albumin. Colloids Surf. B Biointerfaces.

[74] Yano Y.F., Arakawa E., Voegeli W., Kamezawa C., Matsushita T. (2018). Initial Conformation of Adsorbed Proteins at an Air − Water Interface. J. Phys. Chem. B.

[75] Bewersdorff T., Gruber A., Eravci M., Dumbani M., Klinger D., Haase A. (2019). Amphiphilic Nanogels: Influence of Surface Hydrophobicity on Protein Corona, Biocompatibility and Cellular Uptake. Int. J. Nanomed..

[76] Piella J., Bastús N.G., Puntes V. (2017). Size-Dependent Protein-Nanoparticle Interactions in Citrate-Stabilized Gold Nanoparticles: The Emergence of the Protein Corona. Bioconjug. Chem..

[77] Zhang X., Zhang J., Zhang F., Yu S. (2017). Probing the Binding Affinity of Plasma Proteins Adsorbed on Au Nanoparticles. Nanoscale.

[78] Ristroph K.D., Prud’homme R.K. (2019). Hydrophobic Ion Pairing: Encapsulating Small Molecules, Peptides, and Proteins into Nanocarriers. Nanoscale Adv..

[79] Lu J.R., Su T.J., Penfold J. (1999). Adsorption of Serum Albumins at the Air/Water Interface. Langmuir.

[80] Lu J.R., Su T.J., Thomas R.K., Penfold J., Webster J. (1999). Structural Conformation of Lysozyme Layers at the Air/Water Interface Studied by Neutron Reflection. J. Colloid Interface Sci..

[81] Yuan G., Kienzle P.A., Satija S.K. (2020). Salting up and Salting down of Bovine Serum Albumin Layers at the Air-Water Interface. Langmuir.

[82] Lander L.M., Siewierski L.M., Britain W.J., Vogler E.A. (1993). A Systematic Comparison of Contact Angle Methods. Langmuir.

[83] Kumal R.R., Nayak S., Bu W., Uysal A. (2022). Chemical Potential Driven Reorganization of Anions between Stern and Diffuse Layers at the Air/Water Interface. J. Phys. Chem. C.

[84] Cox S.J., Thorpe D.G., Shaffer P.R., Geissler P.L. (2020). Assessing Long-Range Contributions to the Charge Asymmetry of Ion Adsorption at the Air-Water Interface. Chem. Sci..

[85] Jungwirth P., Tobias D.J. (2006). Specific Ion Effects at the Air/Water Interface. Chem. Rev..

[86] Garrett B.C. (2004). Ions at the Air / Water Interface. Science.

[87] Von Grünberg H.H., Mbamala E.C. (2001). Charged Colloids near Interfaces. J. Phys. Condens. Matter.

[88] Mbamala E.C., Von Grünberg H.H. (2002). Effective Interaction of a Charged Colloidal Particle with an Air-Water Interface. J. Phys. Condens. Matter.

[89] Abbina S., Takeuchi L.E., Anilkumar P., Yu K., Rogalski J.C., Shenoi R.A., Constantinescu I., Kizhakkedathu J.N. (2020). Blood Circulation of Soft Nanomaterials Is Governed by Dynamic Remodeling of Protein Opsonins at Nano-Biointerface. Nat. Commun..

[90] Ruh H., Kühl B., Brenner-Weiss G., Hopf C., Diabaté S., Weiss C. (2012). Identification of Serum Proteins Bound to Industrial Nanomaterials. Toxicol. Lett..

[91] Hassan N., Maldonado-Valderrama J., Gunning A.P., Morris V.J., Ruso J.M. (2011). Investigating the Effect of an Arterial Hypertension Drug on the Structural Properties of Plasma Protein. Colloids Surf. B Biointerfaces.

[92] Hernández E.M., Franses E.I. (2003). Adsorption and Surface Tension of Fibrinogen at the Air/Water Interface. Colloids Surf. A Physicochem. Eng. Asp..

[93] Yang L., Biswas M.E., Chen P. (2003). Study of Binding between Protein A and Immunoglobulin G Using a Surface Tension Probe. Biophys. J..

[94] Miller R., Fainerman V.B., Makievski A. (2000). V Dynamics of Protein and Mixed Protein r Surfactant Adsorption Layers at the Water r Fluid Interface. Adv. Colloid Interface Sci..

[95] Tripp B.C., Magda J.J., Andrade J.D. (1995). Adsorption of Globular Proteins at the Air/Water Interface as Measured via Dynamic Surface Tension: Concentration Dependence, Mass-Transfer Consideration, and Adsorption Kinetics. J. Colloid Interface Sci..

[96] Miteva M., Kirkbride K.C., Kilchrist K.V., Werfel T.A., Li H., Nelson C.E., Gupta M.K., Giorgio T.D., Duvall C.L. (2015). Tuning PEGylation of Mixed Micelles to Overcome Intracellular and Systemic SiRNA Delivery Barriers. Biomaterials.

[97] Pelaz B., Del Pino P., Maffre P., Hartmann R., Gallego M., Rivera-Fernández S., De La Fuente J.M., Nienhaus G.U., Parak W.J. (2015). Surface Functionalization of Nanoparticles with Polyethylene Glycol: Effects on Protein Adsorption and Cellular Uptake. ACS Nano.

[98] Ostuni E., Chapman R.G., Holmlin R.E., Takayama S., Whitesides G.M. (2001). A Survey of Structure-Property Relationships of Surfaces That Resist the Adsorption of Protein. Langmuir.

[99] Meesaragandla B., Garcìa I., Biedenweg D., Toro-Mendoza J., Coluzza I., Liz-marza L.M., Delcea M. (2020). H-Bonding-Mediated Binding and Charge Reorganization of Proteins on Gold Nanoparticles. Phys. Chem. Chem. Phys..

[100] Piloni A., Wong C.K., Chen F., Lord M., Walther A., Stenzel M.H. (2019). Surface Roughness Influences the Protein Corona Formation of Glycosylated Nanoparticles and Alter Their Cellular Uptake. Nanoscale.

[101] Gessner A., Waicz R., Lieske A., Paulke B.R., Mäder K., Müller R.H. (2000). Nanoparticles with Decreasing Surface Hydrophobicities: Influence on Plasma Protein Adsorption. Int. J. Pharm..

[102] Chapman R.G., Ostuni E., Takayama S., Holmlin R.E., Yan L., Whitesides G.M. (2000). Surveying for Surfaces That Resist the Adsorption of Proteins. J. Am. Chem. Soc..

[103] Van Niel G., D’Angelo G., Raposo G. (2018). Shedding Light on the Cell Biology of Extracellular Vesicles. Nat. Rev. Mol. Cell Biol..

[104] Buzás E.I., Tóth E., Sódar B.W., Szabó-Taylor K. (2018). Molecular Interactions at the Surface of Extracellular Vesicles. Semin. Immunopathol..

[105] Sódar B.W., Kittel Á., Pálóczi K., Vukman K.V., Osteikoetxea X., Szabó-Taylor K., Németh A., Sperlágh B., Baranyai T., Giricz Z. (2016). Low-Density Lipoprotein Mimics Blood Plasma-Derived Exosomes and Microvesicles during Isolation and Detection. Sci. Rep..

[106] Németh A., Orgovan N., Sódar B.W., Osteikoetxea X., Pálóczi K., Szabó-Taylor K., Vukman K.V., Kittel Á., Turiák L., Wiener Z. (2017). Antibiotic-Induced Release of Small Extracellular Vesicles (Exosomes) with Surface-Associated DNA. Sci. Rep..

[107] Tóth E., Turiák L., Visnovitz T., Cserép C., Mázló A., Sódar B.W., Försönits A.I., Petővári G., Sebestyén A., Komlósi Z. (2021). Formation of a Protein Corona on the Surface of Extracellular Vesicles in Blood Plasma. J. Extracell. Vesicles.

[108] Théry C., Witwer K.W., Aikawa E., Alcaraz M.J., Anderson J.D., Andriantsitohaina R., Antoniou A., Arab T., Archer F., Atkin-Smith G.K. (2018). Minimal Information for Studies of Extracellular Vesicles 2018 (MISEV2018): A Position Statement of the International Society for Extracellular Vesicles and Update of the MISEV2014 Guidelines. J. Extracell. Vesicles.

[109] Liu P., Pearce C.M., Anastasiadi R., Resmini M., Castilla A.M. (2019). Covalently Crosslinked Nanogels: An NMR Study of the Effect of Monomer Reactivity on Composition and Structure. Polymers.

[110] Spivak D., Shea K.J. (1999). Molecular Imprinting of Carboxylic Acids Employing Novel Functional Macroporous Polymers. J. Org. Chem..

