# Supplementary material for: Protein-Nanoparticle Interactions Govern the Interfacial Behavior of Polymeric Nanogels: Study of Protein Corona Formation at the Air/Water Interface
Source: Int J Mol Sci. 2023 Feb 1;24(3):2810. doi: 10.3390/ijms24032810 (PMC9917661; doi:10.3390/ijms24032810)
Supplement: Supplementary file 1 [file ijms-24-02810-s001.zip › ijms-2185897-supplementary.pdf]

Supplementary Materials

Protein-Nanoparticle interactions govern the interfacial behavior of polymeric nanogels: study of protein corona formation at the air/water interface

Federico Traldi,[a] Pengfei Liu,[a] Inês Albino,[b] Lino Ferreira,[b] Ali Zarbakhsh [a] and Marina Resmini.[a]

[a] School of Physical and Chemical Science, Queen Mary University of London, London E1 4NS, UK.

[b] CNC-Center for Neuroscience and Cell Biology, CIBB-Centre for Innovative Biomedicine and Biotechnology, University of Coimbra, UC, Biotech Parque Tecnológico de Cantanhede, 3060-197 Coimbra, Portugal.

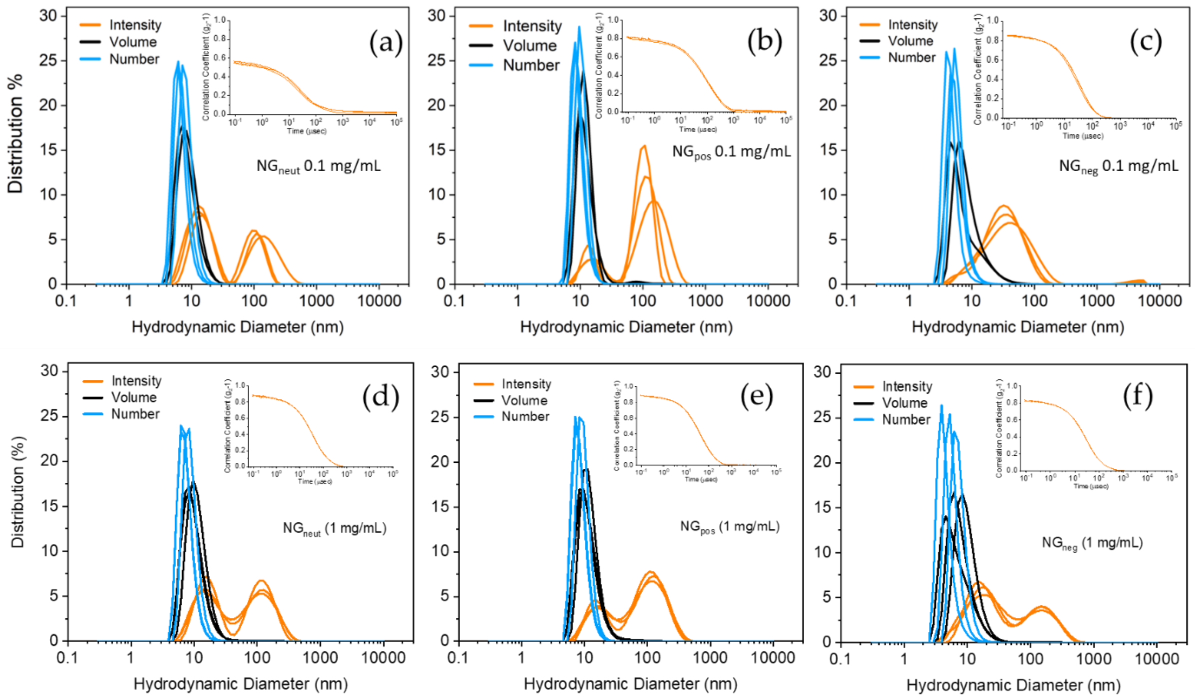

Figure S1. Triplicate DLS measurements of a) NG_{neut}; b) NG_{pos}; and c) NG_{neg} in PBS (100 µg mL^{-1}, 25°C) by intensity (orange), volume (black) and number (blue) distributions. Analysis conducted at 1000 µg mL^{-1} in same conditions is shown for d) NG_{neut}; e) NG_{pos}; and f) NG_{neg}. Inserts show correlogram of each triplicate measurement. Presence of a single peak by volume and number distributions suggests that the second population observed by intensity represents a negligible fraction of the sample.

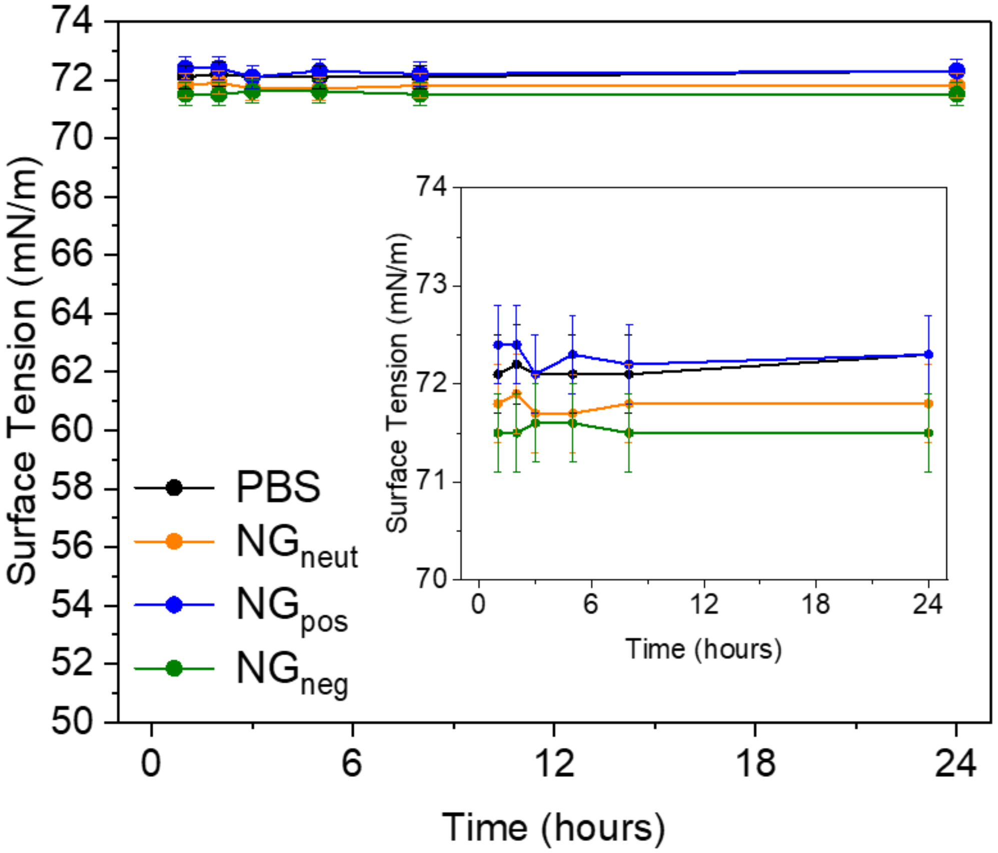

Figure S2. Static surface tension of NGneut (neutral, orange), NGpos (positive, blue) and NGneg (negative, green) measured at 100 µg mL^{-1} in PBS (25°C). Insert shows the same data in a range between 70 and 74 mN/m to better distinguish individual sample lines. Data showed NGs did not deviate from the surface tension of the control PBS (black, 72 ± 0.5 mN/m) over a period of 24h, indicating that nanogels are not surface active.

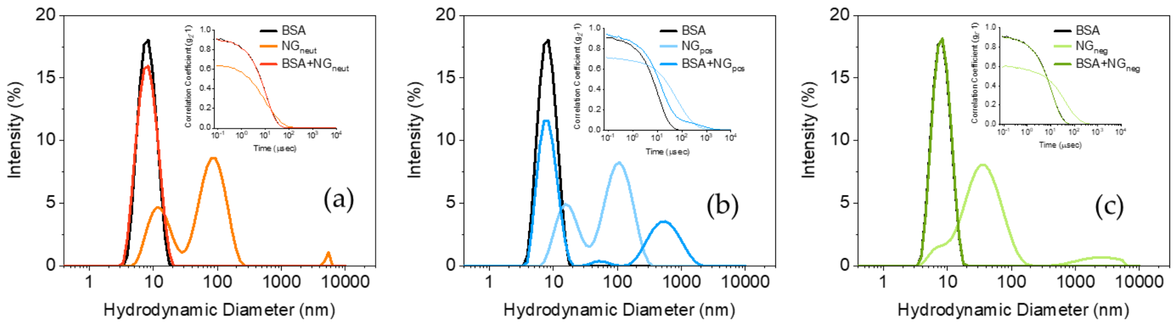

Figure S3. Dynamic light scattering (intensity distribution) of a) neutral NGneut, b) positively charged NGpos, and c) negatively charged NGneg (100 µg mL $^{-1}$) in the presence of BSA (35 mg mL^{-1}) in PBS (10 mM, pH 7.4) at 25 °C. Pure BSA (black) was analysed as control. Inserts

represent correlograms obtained for each analysis. All measurements were obtained in triplicates (single representative run shown here for each sample).

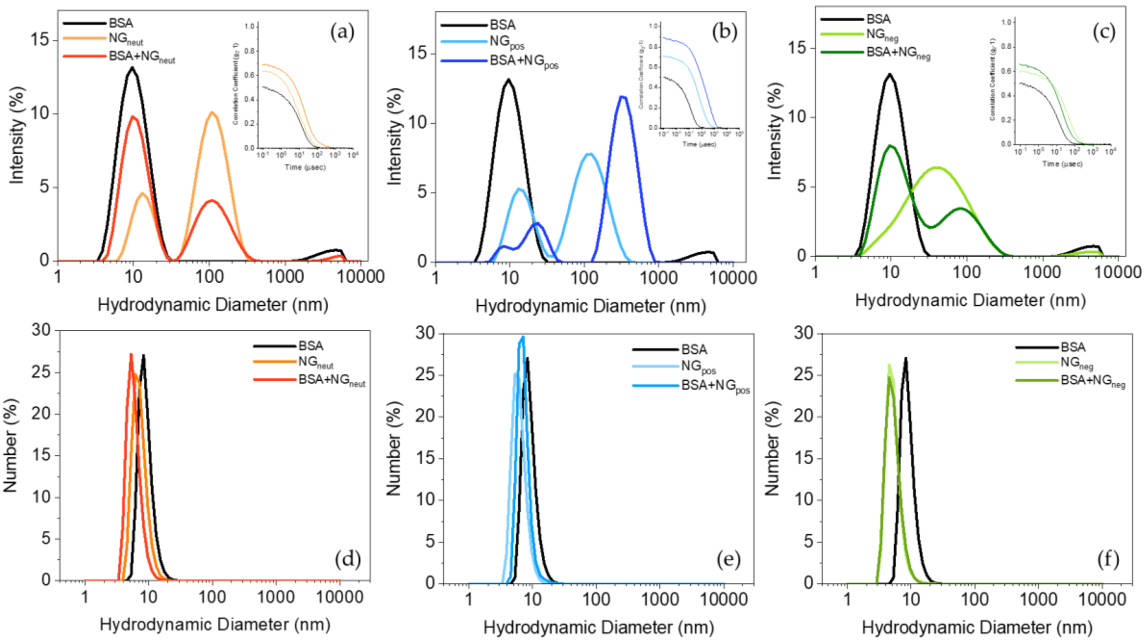

Figure S4. Dynamic light scattering by intensity (a-c) or number (d-f) for neutral NG_{neut} (orange), positively charged NG_{pos} (blue), and negatively charged NG_{neg} (green) (100 μg mL $^{-1}$) in the presence of BSA (100 μg mL $^{-1}$) in PBS (10 mM, pH 7.4) at 25 °C. Pure BSA (black) was analysed as control. Inserts represent correlograms obtained for each analysis. All measurements were obtained in triplicates (single representative run shown here for each sample).

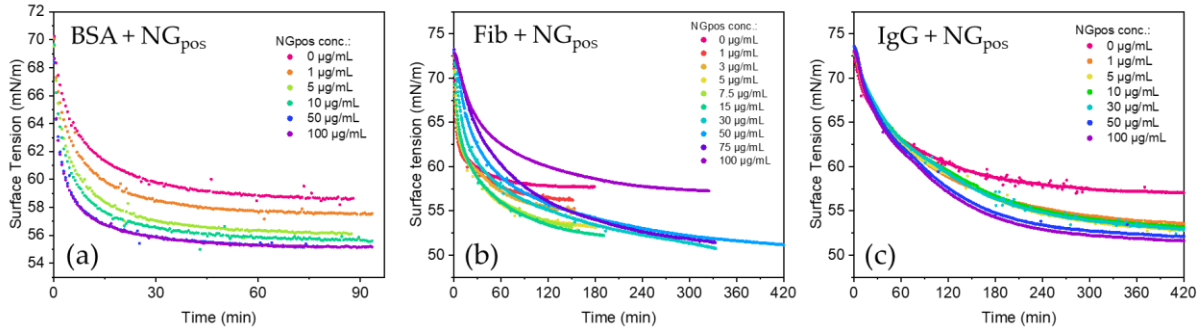

Figure S5. Dynamic surface tensiometry of a) BSA (100 μg mL^{-1}), b) Fib (75 μg mL^{-1}) and c) IgG (100 μg mL^{-1}) as a function of NG_{pos} concentration (shown in the legend) in PBS. All measurements were carried out at 25°C.

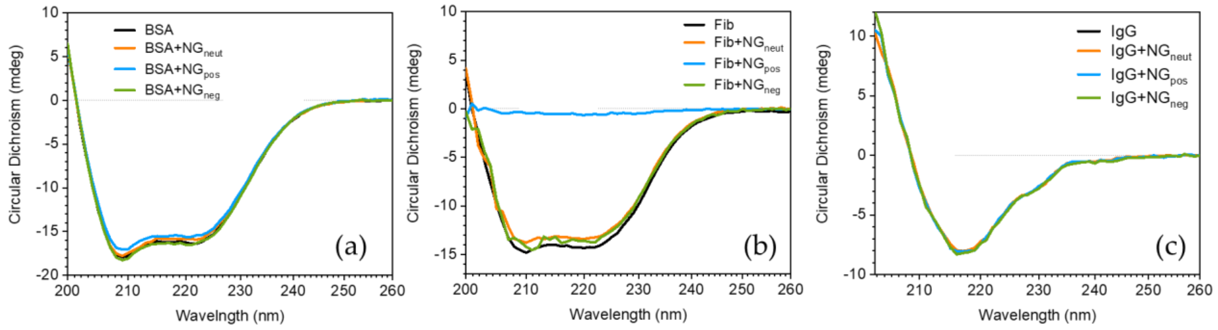

Protein (concentration)	NGneut (µg mL-1)	NGpos (µg mL-1)	NGneg (µg mL-1)
BSA (100 µg mL-1)	100	100	100
Fib (300 µg mL-1)	400	200	400
IgG (400 µg mL-1)	400	200	400

Figure S6. Circular dichroism spectra of a) BSA (100 µg mL-1); b) Fib (300 µg mL-1); and c) IgG (400 µg mL-1) pure (black) or in presence of nanogels in PBS (10 mM, pH 7.4). Bottom table shows concentrations of NGs employed for this study. Concentration of nanogels were adapted to match the relative ratio of $NG\!:\!proteins$ in surface tension experiments. To ensure good correlation between CD and surface tension data, samples were measured immediately after preparation and again after a time equal to the t98%, with no time-dependent variations in the CD spectra observed (data not shown). In the case of NGpos-Fib mixtures (b, blue line), presence of aggregation resulting from the tubular structure of Fib, prevented conclusive evaluation of the secondary structure of the protein.

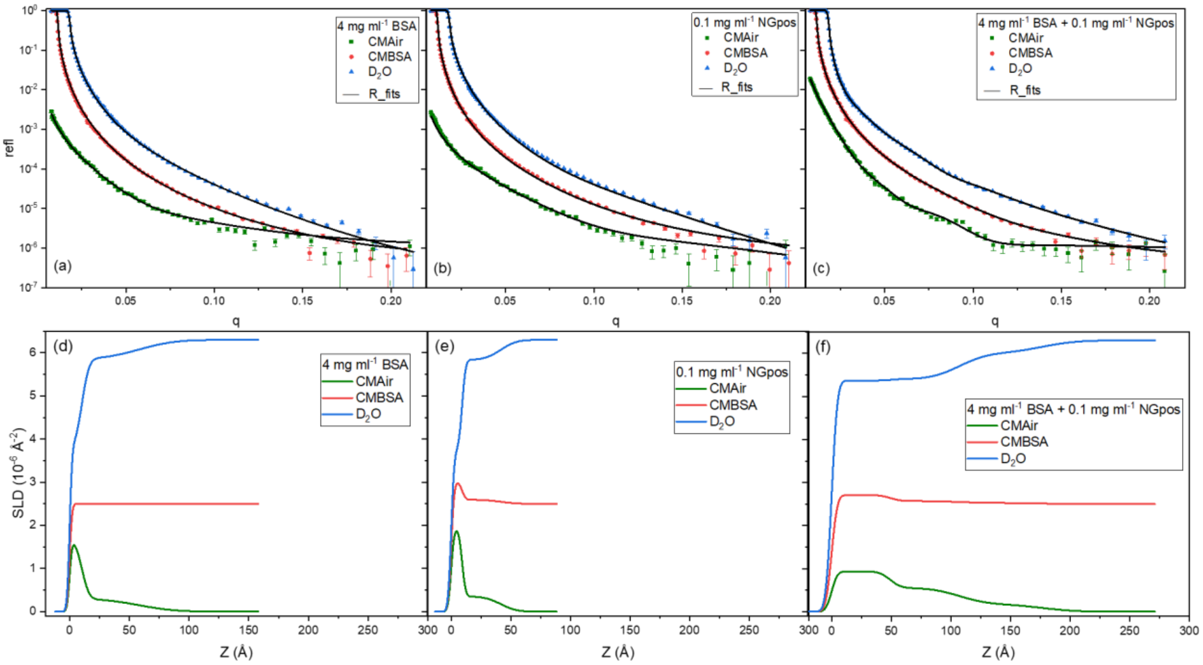

Figure S7. NR data (markers) and fitting (solid lines) of (a) 4 mg ml-1 BSA, (b) 0.1 mg ml-1 NGpos and (c) the mixtures of them at air-PBS buffer interfaces in CMAir, CMBSA and D2O contrasts. Corresponding SLD profiles are shown in (d), (e) and (f), respectively. For CMAir and CMBSA

contrasts, NG_{pos} is made of 60%d3AM-20%MBA-20%GUA whilst for the D2O contrast, NG_{pos} is made of 60%AM-20%MBA-20%GUA.

Table S1. Fitting parameters of 4 mg ml^{-1} BSA, 0.1 mg ml^{-1} NG_{pos} and the mixture of them at air-water interface.

sample	layer	thickness/Å	SLD/10^{-6} Å^{-2}			Solvent/%	roughness/Å
4 mg ml^{-1} BSA	fronting	/	0	0	0	/	/
	1	10.3 ± 0.4	3.3	2.5	2	15.4 ± 3.1	1.7 ± 0.2
	2	47.6 ± 1.2	3.3	2.5	2	85.0 ± 0.4	4.8 ± 0.5
	backing	/	6.3 (D2O)	2.5 (CMBSA)	0 (CMAir)	/	23.8 ± 0.7
0.1 mg ml^{-1} NGpos	fronting	/	0	0	0	/	/
	1	9.2 ± 0.2	1.8	3.4	3.4	43.0 ± 1.0	2.1 ± 0.2
	2	32.3 ± 1.2	1.8	3.4	3.4	89.7 ± 0.6	2.4 ± 0.6
	backing	/	6.3 (D2O)	2.5 (CMBSA)	0 (CMAir)	/	10.5 ± 1.0
4 mg ml-1 BSA + 0.1 mg ml-1 NGpos	fronting	/	0	0	0	/	/
	1	49.1 ± 0.5	5.37 ± 0.01	2.71 ± 0.01	0.93 ± 0.01	/	3.8 ± 0.1
	2	58.1 ± 0.9	5.40 ± 0.01	2.57 ± 0.01	0.55 ± 0.01	/	7.1 ± 1.5
	3	69.4 ± 0.6	6.01 ± 0.01	2.53 ± 0.01	0.18 ± 0.01	/	18.5 ± 0.7
	backing	/	6.3 (D2O)	2.5 (CMBSA)	0 (CMAir)	/	22.4 ± 2.3

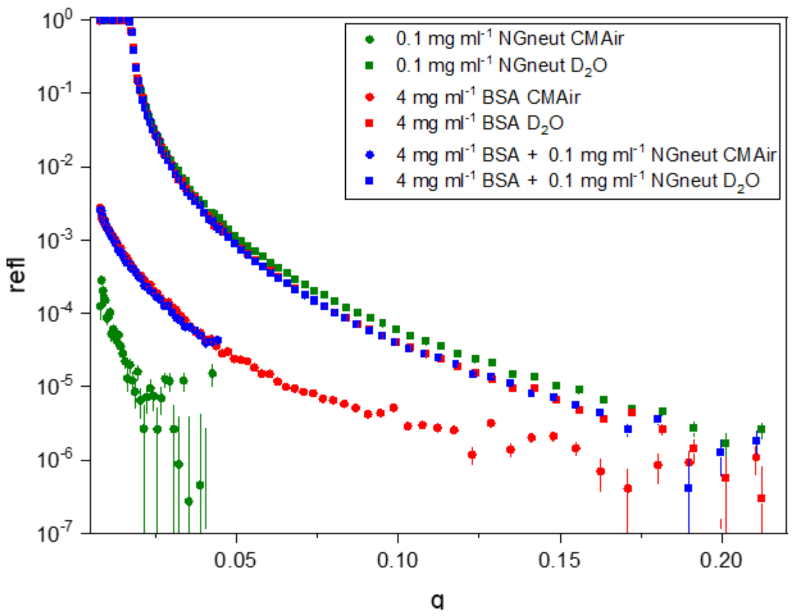

Figure S8. NR data of 0.1 mg ml^{-1} NGneut (green), 4 mg ml^{-1} BSA (red), and the mixtures of them (blue) at air-PBS buffer interfaces in CMAir and D2O contrasts. For CMAir contrast, NGneut is made of 80%d3AM-20%MBA whilst for the D2O contrast, NGneut is made of 80% AM-20%MBA.

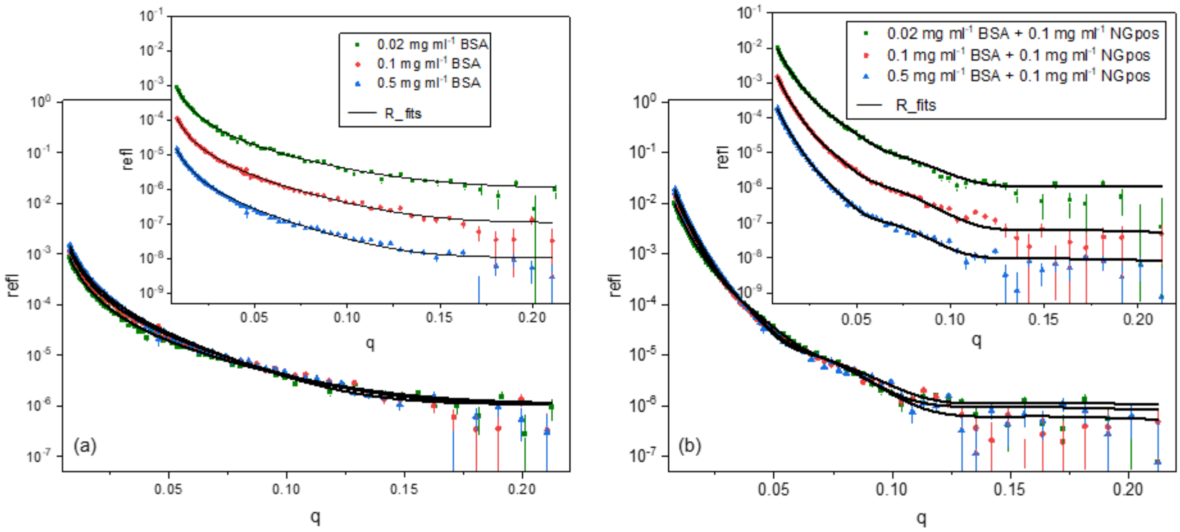

Figure S9. NR data (markers) and fits (solid lines) of (a) BSA and (b) 0.1 mg ml^{-1} NG_{pos}-BSA mixture at different concentrations of BSA at air-PBS buffer interfaces in CMAir water. The inset shows the data which are scaled down by a factor of 10 for clarity.

Table S2. Fitting parameters of BSA solution and BSA-NG_{pos} (0.1 mg ml^{-1}) at different BSA concentrations at air-water interface

sample	layer	thickness/Å	SLD/10^{-6} Å^{-2}	Solvent/%	roughness/Å
0.02 mg ml^{-1} BSA	fronting	/	0	/	/
	1	19.7 ± 1.8	2	53.6 ± 4.2	3.7 ± 1.5
	backing	/	0 (CMAir)	/	6.5 ± 1.7
0.1 mg ml-1 BSA	fronting	/	0	/	/
	1	21.0 ± 1.1	2	48.6 ± 3.2	4.6 ± 2.7
	backing	/	0 (CMAir)	/	9.5 ± 2.5
0.5 mg ml-1 BSA	fronting	/	0	/	/
	1	22.4 ± 1.4	2	47.0 ± 3.6	8.7 ± 1.3
	backing	/	0 (CMAir)	/	8.9 ± 1.3
0.02 mg ml-1 BSA + 0.1 mg ml-1 NGpos	fronting	/	0	/	/
	1	46.7 ± 2.9	0.84 ± 0.03	/	4.5 ± 0.9
	2	51.5 ± 2.4	0.36 ± 0.03	/	6.6 ± 1.6
	3	69.4 ± 0.6	0.08 ± 0.01	/	16.8 ± 2.1
	backing	/	0 (CMAir)	/	26.3 ± 2.2
0.1 mg ml-1 BSA + 0.1 mg ml-1 NGpos	fronting	/	0	/	/
	1	48.1 ± 2.5	0.86 ± 0.01	/	4.5 ± 0.9
	2	59.5 ± 1.3	0.48 ± 0.02	/	6.1 ± 1.2
	3	90.3 ± 3.3	0.13 ± 0.01	/	22.4 ± 2.1
	backing	/	0 (CMAir)	/	36.3 ± 4.4
0.5 mg ml-1 BSA + 0.1 mg ml-1 NGpos	fronting	INF	0	/	/
	1	50.2 ± 1.8	0.88 ± 0.01	/	3.7 ± 1.0
	2	61.1 ± 1.5	0.56 ± 0.03	/	5.4 ± 0.8
	3	87.0 ± 5.7	0.14 ± 0.01	/	22.1 ± 2.2
	backing	INF	0 (CMAir)	/	34.4 ± 4.3

Table S3. Scattering length and scattering length density of BSA and nanogels used in the work

Materials	density g cm^{-3}	Scattering length 10^{-5} Å	Scattering length density 10^{-6} Å^{-2}
BSA[(a)]	1.39	15860 (CMAir)<br>21850 (CMBSA)<br>25730 (D_2O)	2.0<br>2.5<br>3.3
NG_{pos}<br>60%-AM+ 20%MBA-20%GUA	1.34 [(b)]	26.66	1.8
$NG_{pos\text{-}d}$<br>60%d3-AM+ 20%MBA-20%GUA	1.50	45.40	3.4

(a) Data taken from the reference: Journal of Colloid and Interface Science 1999 Vol. 213 Issue 2 Pages 426-437

(b) Dry nanogel density was estimated using an AccuPyc 1330 helium pycnometer.

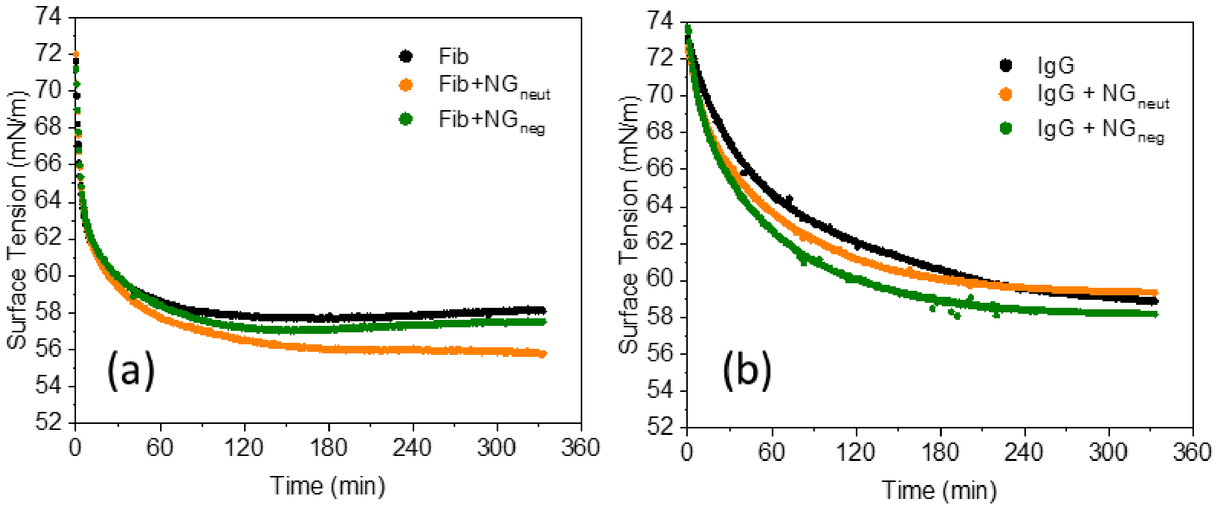

Figure S10. Dynamic surface tensiometry of (NG$_{neut}$) and negatively (NG$_{neg}$) charged nanogels (at concentration of 100 μg mL^{-1}) with a) 75 μg mL^{-1} of Fib and b) 100 μg mL^{-1} of IgG.

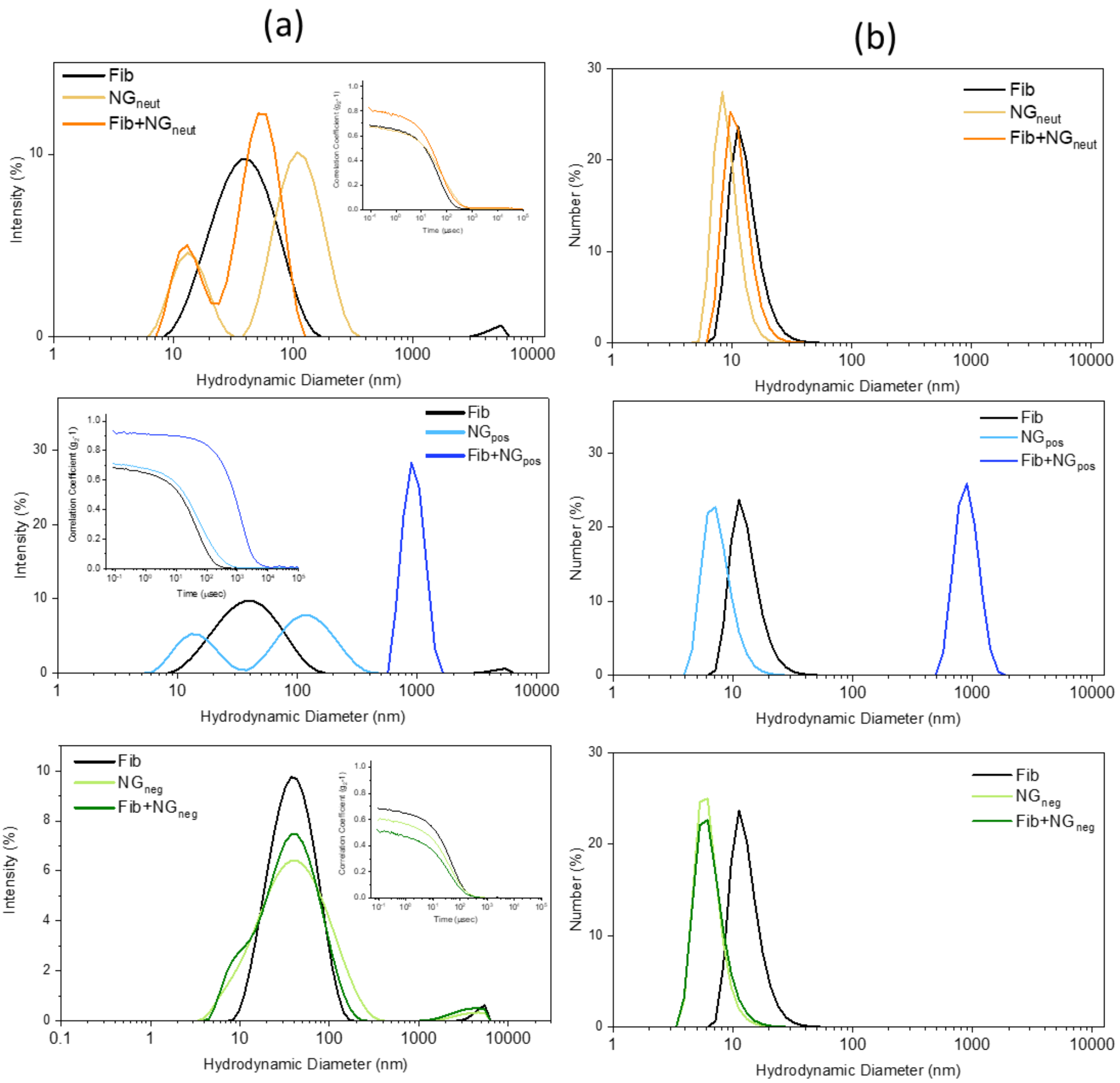

Figure S11. Dynamic light scattering analysis of 100 µg mL^{-1} of neutral NG_{neut} (orange), positively charged NG_{pos} (blue), and negatively charged NG_{neg} (green) in the presence of 75 µg mL^{-1} of human fibrinogen (Fib) by a) intensity and b) number distributions. Number distribution has been included to show a more accurate representation of the relative abundance of the individual populations observed in the results by intensity. Insert in the result by intensity shows the correlogram associated with the analysis. Data show no sign of interaction between Fib and NG_{neut} or NG_{neg}. However formation of large particles upon incubation of Fib with NG_{pos} indicate formation of interactions.

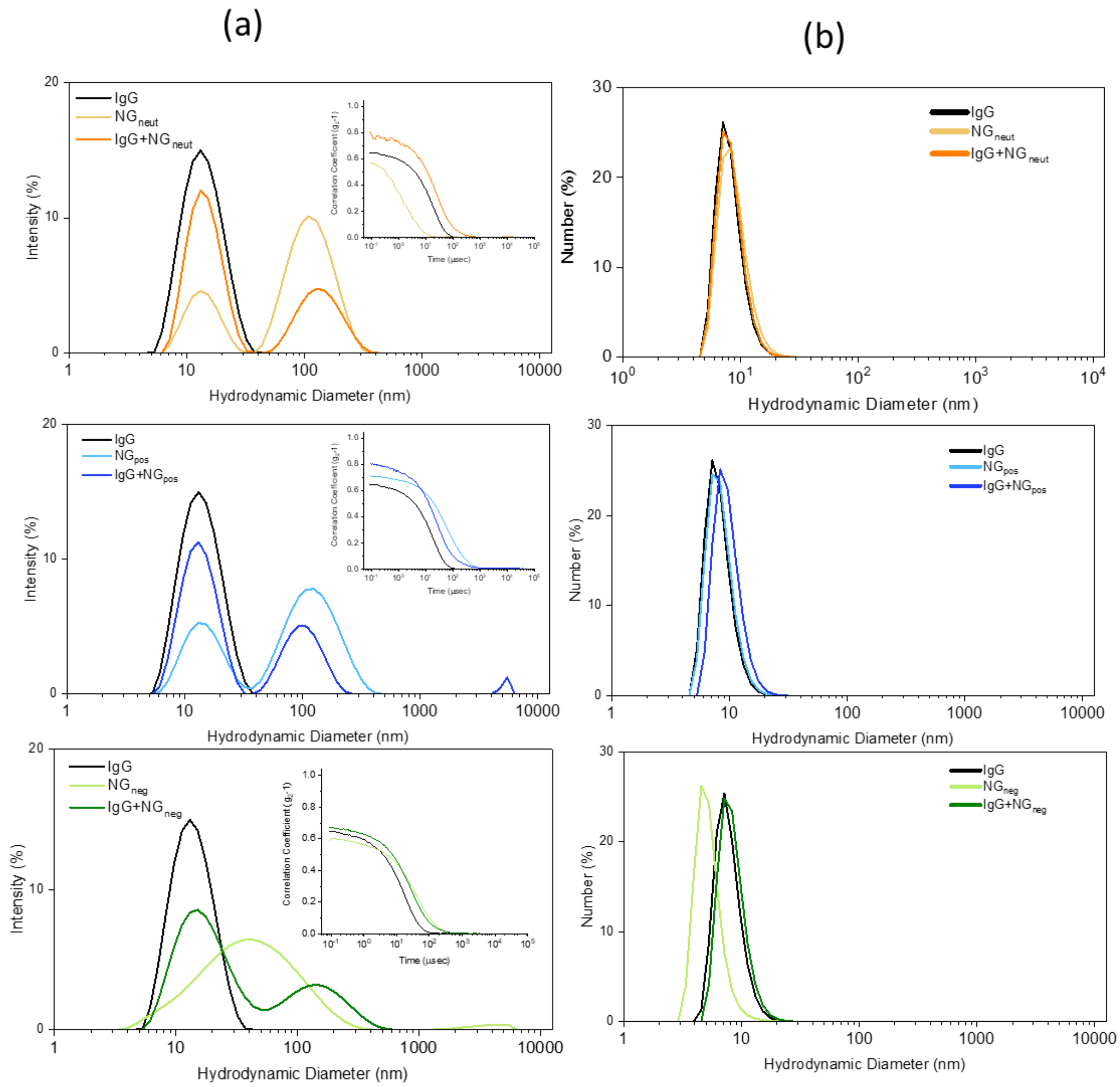

Figure S12. Dynamic light scattering analysis of 100 μg mL^{-1} of neutral NG_{neut} (orange), positively charged NG_{pos} (blue), and negatively charged NG_{neg} (green) in the presence of 100 μg mL^{-1} of IgG by a) intensity and b) number distributions. Number distribution has been included to show a more accurate representation of the relative abundance of the individual populations observed in the results by intensity. Insert in the result by intensity shows the correlogram associated with the analysis. Data show no sign of interaction between IgG and the nanogels, indicating the limitation of using DLS to investigate the protein corona formation of nanoparticles and proteins of similar size.

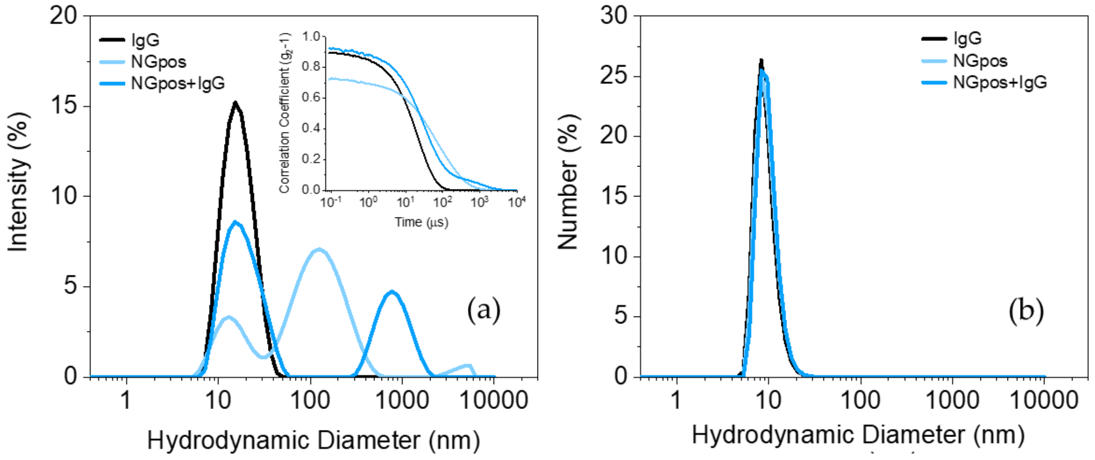

Figure S13. DLS spectra of IgG (black), positively charged NG_{pos} (light blue) and their mixtures (darker blue) by a) intensity or b) number distributions. IgG concentration was 5 mg mL^{-1}, while all nanogels were added at a concentration of 0.1 mg mL^{-1}. Inserts represent relative correlation functions. Concentrations of Fib and IgG simulate levels found in human plasma.

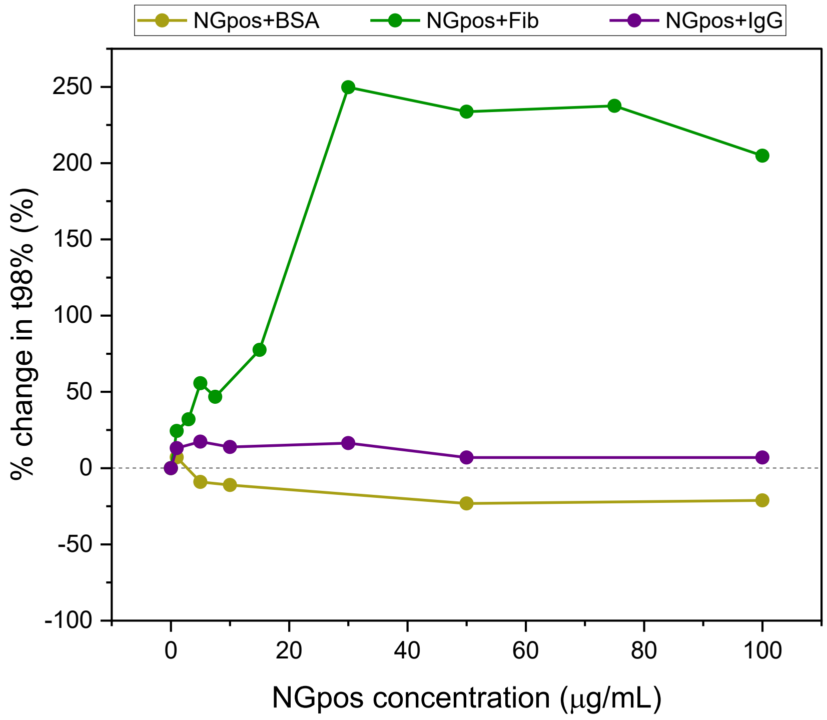

Figure S14. Relative change in equilibration time (t98%) of NG_{pos} mixtures with BSA (yellow), Fib (green) and IgG (purple) as a function of NG_{pos} concentration. NG_{pos} caused shifts <25% in the time of equilibration of BSA and IgG, whereas for Fib increase by > 200% was found, indicating significant increase in size of the nanogel upon completion with Fib.

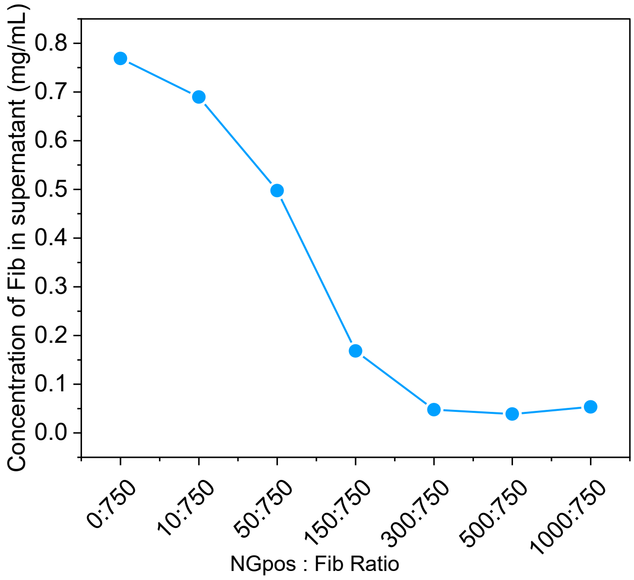

Figure S15. Concentration of free Fib (280 nm) in supernatant as a function of NGpos:Fib ratio after centrifugation and removal of NGpos-Fib complexes. The concentration of Fib in the supernatant reached nearly zero when 300 µg mL-1 of NGpos were added to 750 µg mL-1 of Fib (300:750), indicating almost complete complexation of Fib.

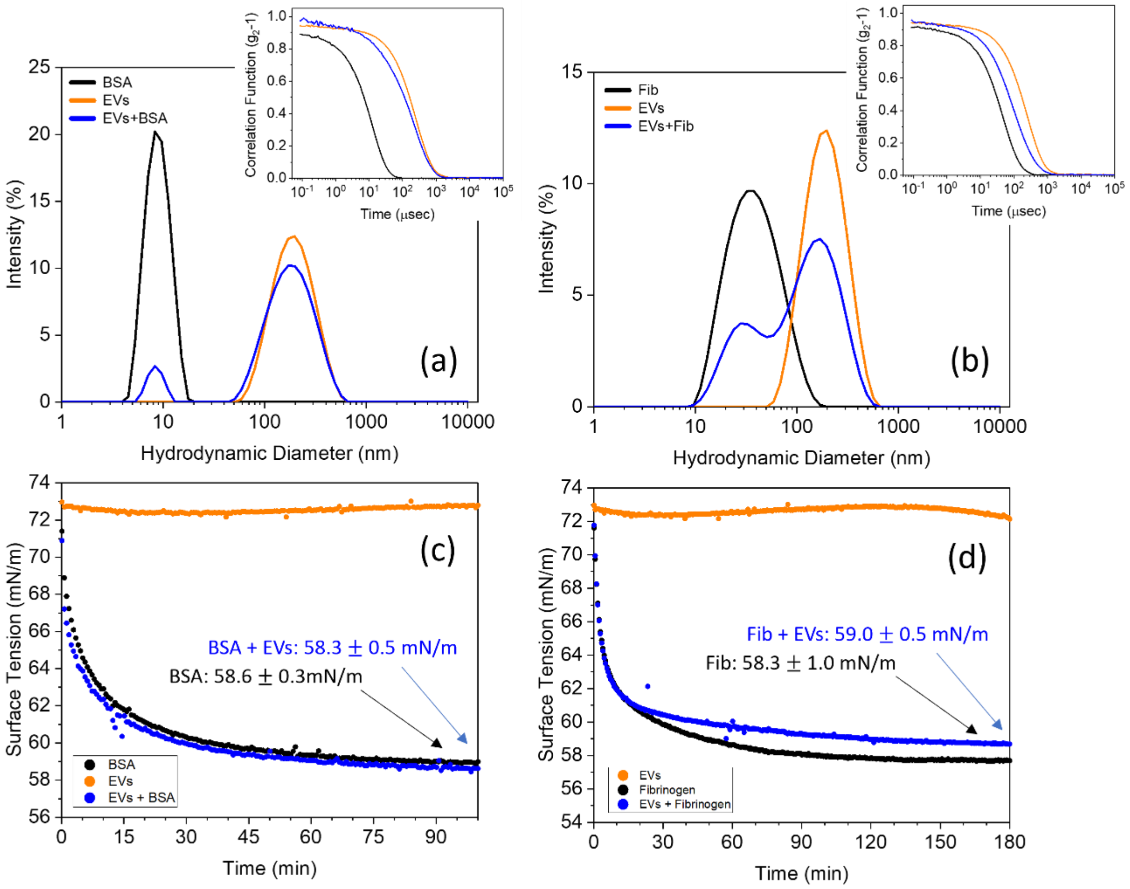

Figure S16. Protein corona study by DLS (a, b) and dynamic surface tensiometry (c, d) of EVs (0.0026 nM), with BSA (100 μg mL^{-1}) and Fib (75 μg mL^{-1}). Both DLS and surface tensiometry showed no significant interactions between proteins and EVs.
